# Integrative miRNOMe profiling reveals the miR‐195‐5p–CHEK1 axis and its impact on luminal breast cancer outcomes

**DOI:** 10.1002/1878-0261.70077

**Published:** 2025-06-23

**Authors:** Veronika Boušková, Marie Ehrlichová, Alžběta Spálenková, Ivona Krus, Simona Šůsová, Viktor Hlaváč, Vlasta Němcová, Renata Koževnikovová, Markéta Trnková, David Vrána, Jiří Gatěk, Kateřina Kopečková, Marcela Mrhalová, Soňa Měšťáková, Pavel Souček

**Affiliations:** ^1^ Toxicogenomics Unit National Institute of Public Health Prague Czech Republic; ^2^ Biomedical Center, Faculty of Medicine in Pilsen Charles University Pilsen Czech Republic; ^3^ Department of Biochemistry, Cell and Molecular Biology, Third Faculty of Medicine Charles University Prague Czech Republic; ^4^ MEDICON a.s. Prague Czech Republic; ^5^ Unilabs Pathology k.s. Prague Czech Republic; ^6^ Comprehensive Cancer Center of Hospital AGEL Novy Jicin Czech Republic; ^7^ EUC Hospital Zlin and Tomas Bata University in Zlin Czech Republic; ^8^ Department of Oncology, Second Faculty of Medicine Charles University and Motol University Hospital Prague Czech Republic; ^9^ Department of Pathology and Molecular Medicine, Second Faculty of Medicine Charles University and Motol University Hospital Prague Czech Republic; ^10^ Department of Surgery, Second Faculty of Medicine Charles University and Motol University Hospital Prague Czech Republic

**Keywords:** breast carcinoma, chemotherapy, DNA damage response, luminal subtype, microRNA, prognosis

## Abstract

The luminal subtype (estrogen receptor‐positive, ER+) is the most common and the most heterogeneous type of breast carcinoma (BC) in women. During our study, we determined expression levels of all microRNAs (miRNome) in 101 ER+ BC samples and identified 25 miRNAs being associated with proliferative markers. Using comprehensive *in silico* analyses we prioritized CHEK1, CDC25A, and CCNE1 as candidate genes affecting the proliferation of ER+ BC, with two microRNAs from the miR‐497∼195 cluster identified as their potential regulators. In a cohort of 217 patients, we found a significant association between high expression of CHEK1 and shorter relapse‐free survival (RFS) in luminal BC patients treated with adjuvant chemotherapy, especially in patients with luminal A subtype. In patients treated with neoadjuvant therapy, the opposite role for RFS was observed for hsa‐miR‐195‐5p. Subsequently, we confirmed the potency of hsa‐miR‐195‐5p to inhibit the expression of CHEK1 *in vitro*. Moreover, the specific Chk1 inhibitor rabusertib (LY2603618) significantly enhanced the efficacy of doxorubicin in both ER+ and ER‐ cell lines. In summary, we have identified the association of a specific miRNA profile with highly proliferative luminal BCs and demonstrated the ability of hsa‐miR‐195‐5p to inhibit CHEK1 expression in BC *in vitro*, underlining the importance of CHEK1 expression and its inhibition for prognosis and treatment of patients with luminal BCs.

## Graphical abstract

In luminal (ER+) breast carcinoma (BC), miRNA profiling identified miR‐195‐5p as a key regulator of proliferation that targets CHEK1, CDC25A, and CCNE1. High *CHEK1* expression correlates with worse relapse‐free survival after chemotherapy, especially in patients with luminal A subtype. miR‐195‐5p inhibits CHEK1 *in vitro*, and CHEK1 inhibition by rabusertib enhances doxorubicin efficacy, highlighting CHEK1 as a prognostic and therapeutic target.

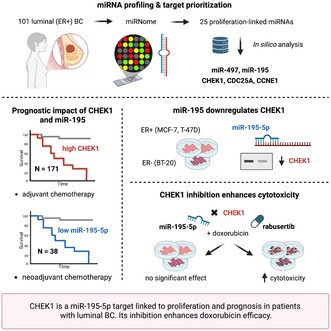

**Abbreviations**
BC: breast carcinoma
B‐H FDR: Benjamini–Hochberg False Discovery Rate
BSA: bovine serum albumin
BTRC: beta‐transducin repeat containing E3 ubiquitin protein ligase
CCND1/3: cyclin D1/3
CCNE1: cyclin E1
CDC25A: cell division cycle 25A
CDK4/6: cyclin‐dependent kinase 4/6
CHEK1/Chk1: checkpoint kinase 1
DDR: DNA damage response
DMSO: dimethyl sulfoxide
E2F3: E2F transcription factor 3
EMT: epithelial‐to‐mesenchymal transition
ER+/−: estrogen receptor‐positive/negative
ERBB2: Erb‐B2 receptor tyrosine kinase 2
FOXK1: Forkhead Box K1
GAD: genetic association database
GAPDH: glyceraldehyde 3‐phosphate dehydrogenase
GEO: gene expression omnibus
HER2: human epidermal growth factor receptor 2
IC50/IC25: inhibitory concentration for 50%/25% of cells
IDC: invasive ductal carcinoma
ILC: invasive lobular carcinoma
KEGG: Kyoto encyclopedia of genes and genomes
Ki‐67: proliferative marker Ki‐67
MCL: Markov cluster algorithm
MEM: minimum essential medium
MIAME: minimum information about a microarray experiment
MIQE: minimum information for publication of quantitative real‐time PCR experiments
miRNA: microRNA
NSCLC: non‐small cell lung cancer
OMIM: online Mendelian inheritance in man
PAM50: 50‐gene prediction analysis of microarrays
PBS: phosphate‐buffered saline
PLK: polo‐like kinase 1
PR: progesterone receptor
RFS: relapse‐free survival
RIN: RNA integrity number
RPMI: Roswell Park Memorial Institute Medium
SDS: sodium dodecyl sulfate
SMAD3/7: SMAD family member 3/7
STR: short tandem repeat
STRING: search tool for the retrieval of interacting genes/proteins
TBS: tris‐buffered saline
TGF‐β: transforming growth factor beta
3′UTR: 3′ untranslated region

## 1 Introduction

The luminal subtype (estrogen receptor‐positive, ER+) is the most common type of breast carcinoma (BC) in women, accounting for approximately 70% of cases [1]. Establishment of intrinsic BC molecular subtypes luminal A and B improved prognostic accuracy and brought therapeutic benefits to patients in clinical practice [2]. This progress was enabled by implementing the 50‐gene Prediction Analysis of Microarrays (PAM50) next to classical clinical–pathological characteristics into the diagnostic process. Subsequently, immunohistochemical determination of hormone receptors expression, overexpression and/or amplification of human epidermal growth factor receptor 2 (ERBB2/HER2, OMIM:164870), and expression of proliferative marker Ki‐67 simplified the BC subtyping [3]. The luminal B subtype differs from the luminal A by generally higher proliferation and a poorer prognosis. Nevertheless, the molecular and clinical heterogeneity within these subtypes is considerable and according to several studies, this variability, expressed, for example, by diverse mutation profiles, copy number variation statuses, epigenetic changes, or by metabolic heterogeneity, might offer the potential for a more personalized approach to the therapy [4, 5, 6, 7].

The treatment of patients with luminal BC is based on hormonal therapy in all patients. For HER2‐positive (HER2+) BC, targeted anti‐HER2 therapy can be included. Chemotherapy can be indicated according to the clinical–pathological characteristics of the tumor, luminal subtype, and clinical condition of the patient. To support a decision on the need for chemotherapy in patients with early stage, HER2‐negative (HER2−) tumors without lymph node involvement, or postmenopausal lymph node‐positive (N1‐3) patients, molecular predictive tests such as Oncotype DX (Genomic Health, Inc., Redwood City, CA), MammaPrint (Agendia, Irvine, CA), Prosigna (PAM50; NanoString Technologies, Seattle, WA), EndoPredict (Myriad Genetics, Inc., Salt Lake City, UT), and IHC4 score could be used [8, 9]. The appropriateness and necessity of chemotherapy are still under study due to the generally lower efficacy of cytotoxic therapy in the luminal subtype, possible severe adverse effects, and the risk of developing resistance to these agents [10].

MicroRNAs (miRNAs) are short (~22 nt) noncoding RNAs involved in a variety of cellular processes, including development, apoptosis, or cell proliferation. Through their complementarity with the 3′UTRs region of their mRNA targets, miRNAs negatively regulate gene expression at the post‐transcriptional level. Changes in miRNA levels were observed in diverse malignancies and were functionally connected with cancer development and progression, including processes associated with metastatic spread and treatment efficacy [11, 12].

To better understand changes in miRNA expression in luminal breast cancer patients and their importance for prognosis and response to chemotherapy, we determined the overall miRNA expression profile in 101 luminal BCs and subsequently identified miRNAs associated with the main pathological proliferative markers. Using different prediction databases and enrichment analysis, we identified CHEK1, CDC25A, and CCNE1 as candidate genes affecting the proliferation of luminal BCs and miR‐195‐5p with miR‐497‐5p as their potential regulators. A larger cohort of 217 patients was used for the evaluation of the clinical importance of *CHEK1* expression in luminal BCs treated with adjuvant chemotherapy. We then confirmed the inhibitory potency of hsa‐miR‐195‐5p towards CHEK1 in ER− and ER+ breast cancer cell lines and explored the ability of specific CHEK1 inhibitors to modulate doxorubicin efficacy *in vitro*.

## 2 Methods

### 2.1 Patients

This study included 217 patients diagnosed with primary BC at the Motol University Hospital, the Institute for the Care for Mother and Child (Prague, Czech Republic), and the Hospital Atlas (Zlin, Czech Republic) between 2003 and 2019. Fresh frozen tumor tissue samples (*N* = 217) and tumor‐adjacent mammary tissue samples (*N* = 18) were collected during surgery as previously described [13], and histological classification of carcinomas was performed according to standard diagnostic procedures [14]. Expression of estrogen (ER) and progesterone (PR) receptors was assessed immunohistochemically with the 1% cut‐off value for classification of carcinomas as hormone receptor‐positive. HER2 (ERBB2, OMIM:164870) status was defined as positive in samples with immunohistochemical score 3+, and 2+ only if confirmed by fluorescence *in situ* hybridization (FISH) or chromogenic *in situ* hybridization (CISH) analysis. Luminal BC subtype was defined by positive immunohistochemical ER status (≥1%). Luminal B subtype was distinguished from luminal A subtype according to overexpression/amplification of HER2, high expression of Ki‐67 antigen (≥14%), or low expression of PR (<20%) [3]. Tumor samples from a subgroup of patients (*N* = 101) were used for primary miRNome profiling. Clinical characteristics of these patients are described in Table S1. All patients were adjuvantly treated with chemotherapy and with subsequent hormonal therapy and eventually anti‐HER2 therapy (in case of HER2 status positive). In the miRNA/mRNA validation study, the original patient cohort was expanded to 217 patients of which 177 (including the original 101 patients) were treated with adjuvant chemotherapy based on combinations of anthracyclines, cyclophosphamide, 5‐fluorouracil, and taxanes and 40 patients treated with neoadjuvant chemotherapy.

Clinical characteristics of the validation cohorts of patients are described in Table 1. The study protocol was approved by the Ethical Commission of the National Institute of Public Health in Prague (approvals no. 9799‐4, NT13679, and NT14055‐3). All patients were informed about the study and those who agreed and signed an informed consent participated in the study. All patient data and procedures complied with the Declaration of Helsinki.

**Table 1:** Clinical characteristics of luminal breast carcinoma patients (*N* = 217) used for validation study.

Characteristics	Adjuvantly treated patients, *N* (%)	Neoadjuvantly treated patients, *N* (%)
Menopausal status		
Premenopausal	39 (22.0)	15 (37.5)
Postmenopausal	127 (71.8)	24 (60.0)
Not available	11 (6.2)	1 (2.5)
Histological type		
Invasive ductal carcinoma	148 (83.6)	34 (85.0)
Invasive lobular carcinoma	21 (11.9)	5 (12.5)
Other types^a^	8 (4.5)	1 (2.5)
Tumor extent (pT)		
pT1	115 (65.0)	21 (52.5)
pT2	56 (31.6)	16 (40.0)
pT3	6 (3.4)	3 (7.5)
Lymph node metastasis (pN)		
Absent (pN0)	71 (40.1)	23 (57.5)
Present (pN1‐3)	104 (58.8)	17 (42.5)
Not available	2 (1.1)	
Pathological TNM stage		
SI	60 (33.9)	16 (40.0)
SII	100 (56.5)	20 (50.0)
SIII	15 (8.5)	4 (10.0)
Not available	2 (1.1)	
Pathological grade		
G1	37 (20.9)	8 (20.0)
G2	99 (55.9)	24 (60.0)
G3	37 (20.9)	8 (20)
GX	4 (2.3)	
Progesterone receptor status		
Positive	146 (82.5)	37 (92.5)
Negative	31 (17.5)	3 (7.5)
HER2 status		
Positive	43 (24.3)	8 (20.0)
Negative	134 (75.7)	32 (80.0)
Ki‐67 expression status		
< 20% (low)	78 (44.1)	15 (37.5)
≥ 20% (high)	85 (48.0)	25 (62.5)
Not available	14 (7.9)	
Subtype		
Luminal A	42 (23.7)	8 (20.0)
Luminal B	131 (74.0)	31 (77.5)
Not available	4 (2.3)	1 (2.5)

a Other types: mixed type (*n* = 9), micropapillary (*n* = 1), and papillary (*n* = 1).

### 2.2 RNA extraction and quality control

Total RNA, including miRNA, was isolated from macrodissected tumor tissues using Trizol Reagent (Invitrogen, CA, USA) and from cell lines by miRNeasy Micro Kit (Qiagen, Hilden, Germany) according to the manufacturer's protocol. Isolated RNA was quantified by Quant‐iT RiboGreen RNA Assay Kit (Invitrogen) using Infinite M200 multiplate reader (Tecan Group Ltd, Männedorf, Switzerland). RNA intergrity (RIN) was checked by Agilent 2100 Bioanalyzer and Agilent RNA 6000 Nano Assay Kit (Agilent Technologies, Inc., CA, USA). The median value and range of RIN of samples used in the study was 6.3, 4.4–9.0 (95.1% of samples with RIN ≥5).

### 2.3 Microarray analysis of miRNA expression

Expression levels of miRNAs were assessed by the SurePrint G3 Human miRNA Microarrays 8 × 60 k, Release 19.0; Design ID 046064 (Agilent Technologies) according to the manufacturers' protocol. MiRNAs in 100 ng of total RNA was labeled with Cy3 using the miRNA Complete Labeling and Hybridization Kit (Agilent Technologies). The Cy3‐labeled samples were hybridized for 20 h at 55 °C in a rotator oven. After washing steps with the Gene Expression Wash Buffer Kit (Agilent Technologies), the array slides were scanned using the Agilent DNA SureScan microarray scanner (Agilent Technologies) and data were extracted from the scanned images using the Feature Extraction version 11.5.1.1 software (Agilent Technologies). The quality criteria predefined by the manufacturer and generated by the Feature extraction software as QC reports were fulfilled for each array. Microarray data was MIAME (Minimum Information About a Microarray Experiment) [15] compliant and has been submitted to the Gene Expression Omnibus (GEO, GSE267543).

### 2.4 Expression analysis of miRNA and mRNA by qPCR

For evaluating miRNA expression levels, cDNA was synthesized from 2 ng of total RNA with 5× RT primers included in TaqMan microRNA Assays and the TaqMan MicroRNA Reverse Transcription Kit (Applied Biosystems, Waltham, MA, USA) according to the manufacturers' protocol. Real‐time PCR quantification (qPCR) was prepared in 5 μL reactions that contained 2.5 μL of 2× TaqMan Universal Master Mix II, 0.25 μL of 20× TaqMan microRNA Assay (Table S2), 0.25 μL of nuclease‐free water, and 2 μL of diluted cDNA. The qPCR was carried out in the ViiA7 Real‐Time PCR System a 384‐well block (Applied Biosystems) with the cycling protocol: 50 °C for 2 min, 95 °C for 10 min, 40 cycles of 95 °C for 15 s, 60 °C for 1 min.

To select optimal reference genes for normalization of miRNA levels in BC, expression levels of four small RNAs (RNU6B, RNU44, RNU48, and U6) and two miRNAs (has‐miR‐16‐5p and hsa‐miR‐23a) were evaluated in 40 tissue samples differing in expression of ER, PR, and HER2 (ER/PR+, HER2−; ER/PR+, HER2+; ER/PR−, HER2+; *n* = 10 in each set). Using geNorm and NormFinder programs [16, 17], miR‐16, RNU48, and U6 were selected as the most stable molecules regardless of BC subtype. The EIF2B1, IPO8, and MRPL19 reference genes were used for normalization of mRNA expression levels as previously described [18].

The preparation of cDNA from mRNA and qPCR quantification of target and reference genes (Table S2) was done as previously described [13]. The qPCR study design adhered to the MIQE Guidelines (Minimum Information for Publication of Quantitative Real‐Time PCR Experiments) [19].

### 2.5 Cell culture

Two human ER+ (MCF‐7, RRID:CVCL_0031; T‐47D, RRID:CVCL_0553) and one ER‐ (BT‐20, RRID:CVCL_0178) BC cell lines were obtained from the CLS (CLS, Eppelheim, Germany) and further maintained in Eagle's MEM (MCF‐7, BT‐20; PAN‐Biotech, Aidenbach, Germany) or in RPMI 1640 medium (T‐47D; PAN‐Biotech) complemented with 1 mm sodium pyruvate (PAN‐Biotech) and 15 mm Hepes (PAN‐Biotech). Culture media were supplemented with 100 U·mL^−1^ penicillin, 100 μg·mL^−1^ streptomycin, and 10% fetal bovine serum (PAN‐Biotech) at 37 °C and under 5% CO_2_ atmosphere. The MycoAlert assay (Lonza, Basel, Switzerland) was regularly used to detect mycoplasma contamination and the cell lines authentication was done using the PowerPlex 16 System (Promega, Thermofisher Scientific) according to the manufacturers' instructions for Short tandem repeat (STR) fragments profiling by the Department of Genetics at the Institute of Criminology (Prague, Czech Republic). The *in vitro* experiments were deployed between 4 and 40 passages except for miRNA mimics transfections, which were performed up to a maximum of 20 passages. All experiments were performed using mycoplasma‐free cell lines.

### 2.6 Transfection of miRNA mimics

MirVana miRNA mimics (hsa‐miR‐195‐5p, ID: MC10827; miR‐1 Positive Control, and Negative Control #1) were purchased from Thermo Fisher Scientific, reconstituted in sterile nuclease‐free water to prepare 20 μm stock solutions, and stored at −20 °C. Working solutions at lower concentrations were freshly prepared by diluting the respective stock solutions in the Opti‐MEM Reduced Serum Media (Thermo Fisher Scientific). The cells (6 × 10^4^ cells or 24 × 10^4^ cells per well for T‐47D and 4 × 10^4^ cells or 16 × 10^4^ cells per well for MCF‐7 and BT‐20) were seeded into 24‐well or 6‐well culture plates and allowed to adhere overnight. A total of 1.5 μL or 6 μL (24‐well or 6‐well plate, respectively) of the Lipofectamine RNAiMAX reagent (Thermo Fisher Scientific) was added to the Opti‐MEM. This mix was then used to prepare final concentrations of 10 nm or 100 nm of miRNA mimic per well. Prepared mixes were incubated for 5 min at room temperature and added to the cells with freshly replaced growth media without antibiotics. To check the efficiency of transfection, the cells were collected after 24 h by trypsinization and centrifuged (1800 rpm, 10 min, 4 °C), and the pellet washed twice with PBS (PAN‐Biotech) for the subsequent RNA and protein extraction. Results are presented as the mean ± SD of at least three independent experiments.

### 2.7 Luciferase vector and assay

Part of the 3′UTR region of the CHEK1 gene, containing a predicted binding site for miR‐195‐5p, SacI and XbaI restriction sites for cloning into a vector, and a NotI restriction site for cloning efficiency control, was purchased as gBlocks from Integrated DNA Technologies (IDT, Coralville, Iowa, USA). The oligonucleotides were digested with SacI‐HF and XbaI (NEB, Ipswich, Massachusetts, USA) and ligated into the pmirGLO vector (Promega Corporation, Madison, WI, USA) at the multiple cloning sites immediately downstream of the luciferase gene. Escherichia coli DH5α (Thermo Fisher Scientific) was used for cloning with standard heat shock transformation protocol. PCR amplification and digestion with NotI‐HF (NEB) were used to verify prepared constructs.

For the luciferase reporter assay, MCF‐7 cells were seeded in 96‐well culture plates (5 × 10^3^), grown overnight, transfected with 100 nm of miRNA mimic using Lipofectamine RNAiMAX, and after 24 h, transfected with 100 ng of the pmirGLO construct or an empty vector as a control using Viafect transfection reagent (Promega Corporation). Firefly and Renilla luciferase activities were measured with the Tecan Infinite M200 plate reader (Tecan Group Ltd.) using the Dual‐Luciferase Reporter System (Promega Corporation) according to the manufacturer's instructions. Results are presented as the mean ± SD of three independent biological replicates.

### 2.8 Western blot analysis

The cell pellets were resuspended and lysed in RIPA buffer (3% Triton X‐100, 10 mm HEPES, pH 7.4, 0.15 m NaCl, 5 mm EDTA) with cOmplete ULTRA and PhosSTOP Tablets (Roche, Basel, Switzerland). The quantification of total protein in the lysates was done using the Pierce BCA Protein Assay Kit (Thermo Fisher Scientific). A 10% (w/v) polyacrylamide gel was loaded with 15 μg of total protein, which was subsequently separated by electrophoresis in the presence of SDS and deposited onto a nitrocellulose membrane. Thermo Fisher Scientific's Blocker™ BLOTTO or 5% BSA in TBS was used to block the membranes for 60 min. Next, primary antibodies diluted in the blocking buffer were incubated overnight at 4 °C. Finally, TBS containing 0.1% Tween®20 (v/v) was used to rinse the membranes, and secondary antibodies were incubated for 60 min at room temperature. We identified CHK1 (1:1000, mouse monoclonal antibody, 2G1D5, Novus Biologicals, Centennial, CO, USA), CCNE1 (1:200, mouse monoclonal antibody, CCNE1/2460, Novus Biologicals), CDC25A (1:500, mouse monoclonal antibody, #336445, R&D Systems, Minneapolis, MN, USA), ERα (1:000, rabbit monoclonal antibody, #8644, Cell Signaling, Danvers, Massachusetts, USA), cleaved caspase‐3 (1:500, rabbit monoclonal antibody, #9664, Cell Signaling), and cleaved PARP (1:750, rabbit polyclonal antibody, #9542, Cell Signaling). Rabbit monoclonal antibody against GAPDH (1:1000, 14C10, Cell Signaling) was used as a reference protein. IRDye® 800CW goat anti‐mouse and IRDye® 680RD goat anti‐rabbit secondary antibodies were used for the detection. The Odyssey® Fc Imaging System (Li‐cor, Lincoln, Nebraska, USA) was used to visualize the proteins, and Image Studio version 4.0.21 (Li‐cor) was used to quantify them. In the case of cleaved caspase and PARP products, the anti‐rabbit horseradish peroxidase‐conjugated secondary antibody (1:10000; Proteintech, Rosemont, Illinois, USA) was used as the secondary antibody. Protein bands were visualized with an enhanced chemiluminescence detection system (Thermo Fisher Scientific) using the Alliance Q9 Chemiluminescence Imager (Uvitec Ltd, Cambridge, England, UK).

### 2.9 Cell viability assays

Doxorubicin and rabusertib (LY2603618) were purchased from Merck (Darmstadt, Germany), diluted in DMSO to 80 mm and 50 mm stock concentrations, respectively, and stored at −20 °C. The cells (5 × 10^3^) were seeded in 96‐well culture plates, grown overnight, and treated for 24 h with doxorubicin (9‐point dilution series from 0.3 nm to 300 μm) or rabusertib (12‐point dilution series from 10 nm to 200 μm). The cellular viability was determined after 24 h treatment using the CellTiter‐Blue Cell Viability Assay (Thermo Fisher Scientific) according to the manufacturer's protocol. Fluorescence intensity was analyzed with the plate reader Tecan Infinite M200 (Tecan Group Ltd.). The IC_50_ and IC_25_ concentrations were determined by the GraphPad Prism 6.0 software. Values are displayed as the mean ± SD of three independent experiments.

The effect of miRNA mimics or rabusertib pretreatment on doxorubicin‐induced cytotoxicity was determined as follows. The 5 × 10^3^ cells were seeded in 96‐well culture plates, grown overnight, and treated with 100 nm of miRNA mimics and 0.3 μL Lipofectamine RNAiMAX per well or 1/10/15 μm of rabusertib for 24 h and subsequently with 10/25 μm of doxorubicin for the next 24 h. The cell viability was determined as described above.

### 2.10 Data analysis and statistics

Total gene signal for all miRNA genes on the microarrays was extracted from the Feature extraction reports, quantile normalized, and log_2_ transformed with the help of AgiMicroRna and Linear Models for Microarray Data (limma) R/Bioconductor software packages (v3.17). The genes detected in <75% of samples or having intensities <20 percentile were excluded from further analysis.

Differences in expression of miRNAs detected by microarrays between studied groups were analyzed with the limma model, and multiple hypothesis testing correction was performed for each comparison using the Benjamini–Hochberg false discovery rate test (B‐H FDR) [20]. Associations of transcript levels obtained by qPCR with clinical data were analyzed by non‐parametric tests (Kruskal–Wallis, Mann–Whitney, and Spearman rank) using ΔCt values (Ct target gene—Ct reference genes, geometric mean) and the SPSS software v16.0 (IBM, Armonk, NY, USA). The tested clinical and pathological variables were as follows: menopausal status (premenopausal vs. postmenopausal), histological type (ductal vs. other types), tumor extent (pT1 vs. pT2 or pT3), lymph node metastasis (pN0 vs. pN1–3), stage (SI vs. SII–SIII), pathological grade (G1 vs. G2 or G3), PR and HER2 expression (positive vs. negative), Ki‐67 expression (low vs. high), and luminal subtype (luminal B vs. luminal A). Statistically significant changes were considered if the B‐H FDR corrected *P*‐value (*P*_adj_) was ≤0.05 with a fold change ≥1.5. The Survminer (v0.4.9) package was used to perform the Kaplan–Meier survival analysis separately for patients treated with adjuvant therapy, those treated with neoadjuvant therapy, and then similarly for luminal A and luminal B patients. Log‐rank and Breslow tests were used to compare survival between normalized gene expression levels divided by quartiles; Q1 indicates the lowest expression and Q4 the highest expression.

*In vitro* data were evaluated by unpaired Student's t‐test (*P < 0.05, **P < 0.01, ***P < 0.001). Fold changes for gene expression levels in treated and control groups were compared with the 2^−ΔΔCT^ method [21]. All *in vitro* experiments were performed in three independent replicates and data are presented as mean ± SD.

For prediction of miRNA target genes, the DIANA micro‐T (v2023) [22], mirMap (v202203) [23], and miRWalk (v3) [24] databases were used with the default settings. Genetic Association Database (GAD) and Online Mendelian inheritance in man (OMIM) databases in the Database for Annotation, Visualization, and Integrated Discovery (DAVID, v2023q3) [25] were used to prioritize genes associated with BC using the keywords “breast cancer”, “breast neoplasms,” or “mammary neoplasms”. We also utilized the UP_tissue database in the DAVID to identify additional genes, irrespective of their association with BC, but with positive expression in normal or cancerous breast tissue, using the keywords “breast” and “mamma”. The Search Tool for the Retrieval of Interacting Genes/Proteins database (STRING, v12.0) [26] was utilized for construction and visualization of a protein–protein interactions using default settings.

## 3 Results

### 3.1 miRNA expression profile significantly associates with the grade and subtype of breast carcinomas

The normalized expression data of 2006 miRNAs covered by microarray in 101 luminal BCs are in Table S3. After filtering out the unexpressed miRNAs or miRNAs with very low expression, an average of 343 genes were detected (from 315 to 346) per sample.

**Fig. 1:**
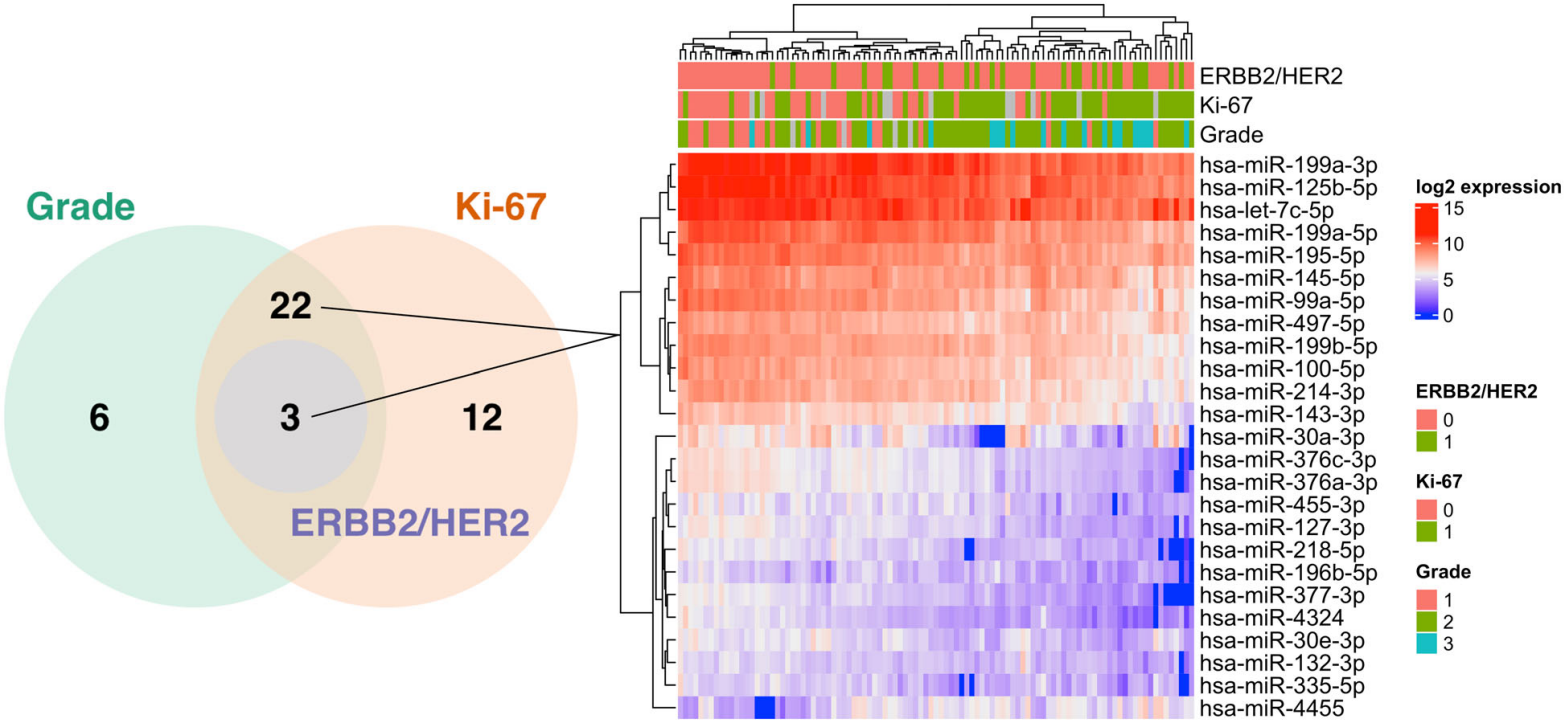
Significantly associated miRNAs with highly proliferative luminal breast carcinomas characterized by high grade and Ki‐67 expression. A total of 25 miRNAs were deregulated simultaneously in tumors with high grade and high expression of Ki‐67 (on the left) and their log_2_ expression levels are shown in the figure on the right. Samples with immunohistochemical score 3+, and 2+ only if confirmed by fluorescence *in situ* hybridization (FISH) or chromogenic *in situ* hybridization (CISH), were considered HER2/ERBB2 positive and those with Ki‐67 expression ≥14% were classified as high (both encoded 1).

Expression profiles of these miRNAs were further analyzed in context with clinical–pathological data. Six miRNAs were upregulated and 25 downregulated in tumors with grade 2 or 3 in comparison to tumors with grade 1 (Table S4). Similarly, expression levels of three miRNAs were upregulated, and 34 miRNAs were downregulated in tumors with high expression of proliferation marker Ki‐67 (≥14%) in comparison to the rest of tumors with low expression (Table S5). Overall, 25 miRNAs were deregulated (hsa‐miR‐4455 upregulated and the rest downregulated) in highly proliferative luminal tumors, which were characterized simultaneously by high grade and high expression of Ki‐67 (Fig. 1). In addition, the expression of three of those miRNAs, namely hsa‐miR‐195‐5p, hsa‐miR‐497‐5p, and hsa‐miR‐132‐3p, was significantly associated with HER2 status (Table S6). We found no significant changes (*P*_adj_ >0.05, fold change <1.5) in miRNA expression between invasive ductal in comparison to other histological types of carcinoma, pT2/pT3 vs. pT1, tumors with lymph nodes metastasis compared to carcinomas without regional metastatic spread, stage SII/SIII vs. SI, or between PR+ and PR‐ tumors. In luminal subtypes, four miRNAs were significantly upregulated and 22 downregulated in luminal B compared to luminal A tumors, and 18 of those miRNAs were also associated with tumor grade and Ki‐67 expression (Table S7).

### 3.2 *Ex vivo* and *in silico* analyses prioritize CHEK1, CDC25A, and CCNE1 as the main clinically associated miRNA targets

Next, we used three databases to predict potential targets of the 25 miRNAs associated in previous analyses with tumor grade and Ki‐67 expression and thus to find possible proliferation‐related genes in luminal BC. With the help of the DIANA micro‐T, mirMap, and miRWalk databases we found 8494, 9750, and 12 680 potential target genes of these 25 miRNAs, respectively. From these gene sets, we specifically chose genes predicted as potential targets of two or more miRNAs from the set of 25 miRNAs, resulting in 4755 genes for DIANA micro‐T, 6182 genes for mirMap, and 8815 genes predicted by miRWalk. Finally, we selected 2065 genes that were predicted as targets by all three databases.

In the subsequent analysis, we excluded genes lacking both ENSEMBL and ENTREZ IDs, utilizing the set of 2058 genes (Table S8). KEGG pathway enrichment analysis suggested that these genes are significantly (B‐H FDR <0.05) enriched in 109 pathways (Table S9). With the help of GAD and OMIM disease databases and tissue expression database, we prioritized 200 genes associated with BC and/or expressed in normal mammary or BC tissue (Table S10). The STRING protein network analysis (highest confidence 0.9; isolated genes removed) with subsequent Markov clustering (MCL) proposed 30 clusters created by two or more proteins containing 84 from 200 genes inserted (Fig. S1). Based on the results of cluster analysis or potential significance for BC (e.g., [27, 28]), we selected ACVR2A, CCNDs, CCNE1, CDC25A, CDK6, CDKN1B, GHR, CHEK1, IGF1R, IRS1, MAPK1, NCOR, PIK3CA, PIK3CB, RARB, SMAD3, SMAD7, and WEE1 for further determination in tumor samples.

**Fig. 2:**
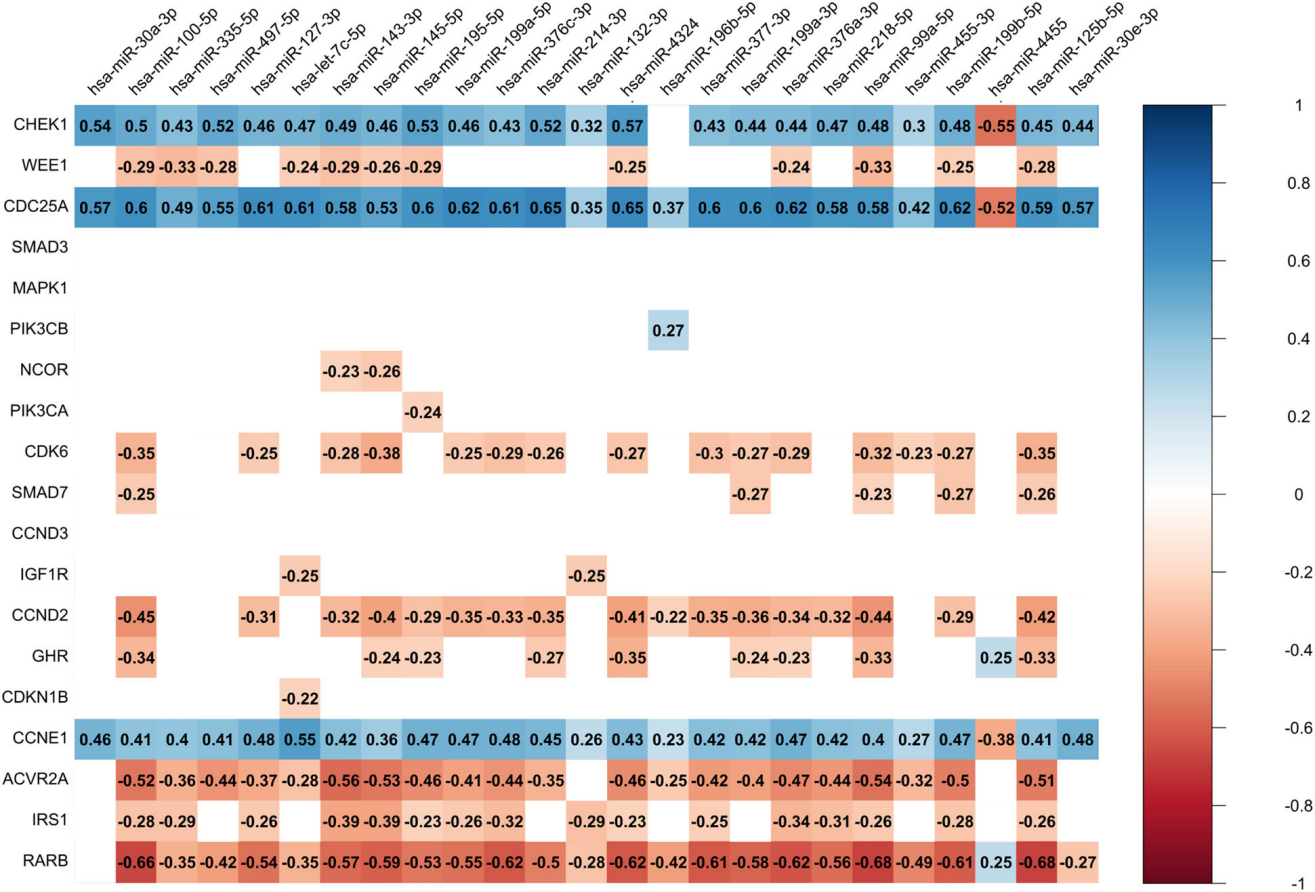
Correlation matrix for miRNAs significantly associated with highly proliferative luminal breast carcinomas identified by miRNome analysis and their target genes. Red represents positive correlation and blue represents negative correlation (Spearman's rank correlation coefficient) as for mRNA (qPCR), higher values correspond to lower expression levels, whereas for miRNA (miRNome), higher values indicate higher expression levels. The white cells depict statistically insignificant correlations (*P* ≥ 0.05, Spearman's rank correlation).

As the next step, we measured the expression levels of these genes in tumor samples of 79 patients previously used for the miRNome analysis. The genes mutually positively correlated with each other, with the most robust associations observed among CHEK1, CDC25A, and CCNE1. ACVR2, PIK3CA, and SMAD7 displayed the highest number of positive correlations (Fig. S2). To enhance the precision in identifying the target genes of the 25 miRNAs linked to highly proliferative luminal BCs, we correlated their expression levels with the 19 selected genes (Fig. 2). Expression levels of only three genes, namely CHEK1, CDC25A, and CCNE1, negatively correlated with the majority of the 25 miRNAs indicating a potential regulatory relationship. According to prediction databases, the DIANA micro‐T, mirMap, and miRWalk, 13 miRNAs were predicted for CHEK1, CDC25A, or CCNE1; specifically, hsa‐miR‐100‐5p, hsa‐miR‐199a‐5p, and hsa‐miR‐199b‐5p (predicted by two databases), as well as hsa‐miR‐127‐3p, hsa‐miR‐132‐3p, hsa‐miR‐196b‐5p, hsa‐miR‐214‐3p, hsa‐miR‐218‐5p, hsa‐miR‐30a‐3p, hsa‐miR‐30e‐3p, and hsa‐miR‐99a‐5p (predicted by one database). Notably, hsa‐miR‐195‐5p and hsa‐miR‐497‐5p were identified as single miRNAs predicted to regulate the expression of all three genes.

As part of the co‐expression analyses, we also identified several positive miRNA‐mRNA correlations, particularly for the ACVR2A, IRS1, and RARB genes (Fig. 2). Therefore, we also analyzed the levels of all genes positively correlated with any of the 25 studied miRNAs in the context of clinical data. High levels of RARB were detected in tumors of patients with grade 1 compared to higher grades, high levels of CCND2 and NCOR were found in tumors of patients with positive involvement of regional lymph nodes, and high levels of CDKN1B were significantly associated with negative expression/amplification of HER2 (Table S11). Considering the positive correlation and the unlikely direct functional connection, we subsequently focused on the CHEK1, CDC25A, CCNE1, hsa‐miR‐195‐5p, and hsa‐miR‐497‐5p genes for further analysis.

### 3.3 Prioritized miRNA gene targets have prognostic value for luminal breast carcinomas

We examined the expression levels and clinical relevance of CHEK1, CDC25A, CCNE1, hsa‐miR‐195‐5p, and hsa‐miR‐497‐5p in a comprehensive cohort of 217 breast carcinoma patients. Specifically, we analyzed BC tissues from 177 patients who underwent adjuvant chemotherapy and 40 patients who received neoadjuvant chemotherapy (Table 1). Additionally, 18 samples of adjacent mammary tissue were included in the analysis. These mammary tissues were collected from patients, mostly diagnosed with invasive ductal or lobular carcinoma (IDC = 13 patients, ILC = 3, invasive papillary or micropapillary carcinoma = 2) as well as the luminal B subtype (LB = 12, LA = 6). The distribution of histological types and luminal subtypes did not differ significantly from the compared tumor tissue cohort (*P* = 0.23, *P* = 0.4, respectively, Fisher's exact test). All these patients belonged to a subgroup that received adjuvant chemotherapy and hormonal treatment following tumor and adjacent tissue removal.

Both evaluated miRNAs exhibited significant downregulation in breast carcinomas compared to adjacent mammary tissues and, in contrast, the expression of CHEK1, CDC25A and CCNE1 genes was significantly increased in malignant versus adjacent tissues (*N* = 18, *P* < 0.01 for all, Table S12). We have confirmed associations of hsa‐miR‐195‐5p and hsa‐miR‐497‐5p with tumor grade, Ki‐67 expression, HER2 status, and luminal subtype in patients treated with adjuvant therapy (*N* = 177, Table S13). Additionally, the expression levels of these two miRNAs were associated with tumor size (pT) and the expression levels of hsa‐miR‐195‐5p with PR status. In the case of CHEK1, CDC25A, and CCNE1, we observed contrasting associations compared to hsa‐miR‐195‐5p and hsa‐miR‐497‐5p with the clinical data, where elevated levels of these genes were linked to high‐grade carcinomas, increased expression of Ki‐67, and (except for CHEK1) were associated with the HER2 and PR‐negative status. High expression levels of these genes were also associated with the luminal B subtype and negative involvement of the regional lymph nodes.

**Fig. 3:**
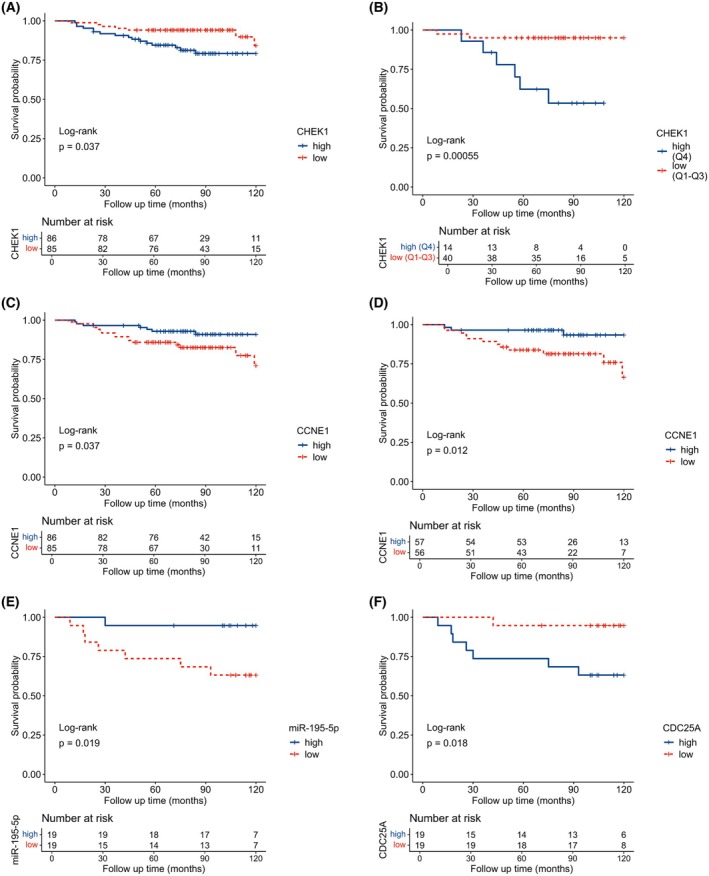
Relapse‐free survival probabilities for breast carcinoma patients stratified by subtype, therapy, and expression of studied genes. Relapse‐free survival (RFS) of adjuvant chemotherapy‐treated patients with luminal subtypes (*N* = 171) (A) or solely the patients with the luminal A subtype (*N* = 54) (B) stratified by the CHEK1 gene expression. RFS of adjuvant chemotherapy‐treated patients with luminal subtypes (*N* = 171) (C) and solely the patients with the luminal B subtype (*N* = 113) (D) stratified by the CCNE1 gene expression. Neoadjuvant chemotherapy‐treated patients with luminal subtypes (*N* = 38) stratified by miR‐195‐5p (E) and CDC25A (F) gene expression levels. The optimal cut‐off for dividing patients with low vs. high expression was the 50th percentile (Q1‐Q2 vs Q3‐Q4) unless otherwise specified.

High CHEK1 expression was associated with shorter RFS in all patients treated with adjuvant chemotherapy (log‐rank: *P* = 0.037, Breslow: *P* = 0.032; Fig. 3A), and the association was more significant in patients with luminal A subtype. The patients in the highest quartile group (Q4) for CHEK1 expression had shorter RFS compared to patients in quartiles 1 through 3 (Q1‐Q3) (log‐rank: *P* < 0.001, Breslow: *P* = 0.001; Fig. 3B). On the contrary, low CCNE1 expression was associated with shorter RFS in the patients unselected for subtype treated with adjuvant chemotherapy (log‐rank: *P* = 0.037, Breslow: *P* = 0.066; Fig. 3C) and this association was more significant in patients with luminal B subtype (log‐rank: *P* = 0.012, Breslow: *P* = 0.021; Fig. 3D). In the patients treated with neoadjuvant chemotherapy, we have seen shorter RFS in patients with high CDC25A expression levels (log‐rank: *P* = 0.018, Breslow: *P* = 0.017) (Fig. 3E), while high hsa‐miR‐195‐5p levels were associated with prolonged RFS in these patients (log‐rank: *P* = 0.019, Breslow: *P* = 0.02) (Fig. 3F). The expression of none of these genes was significantly associated with overall survival of patients in the studied groups (Fig. S3).

### 3.4 miR‐195‐5p represses CHEK1 and CDC25A gene expression *in vitro*

**Fig. 4:**
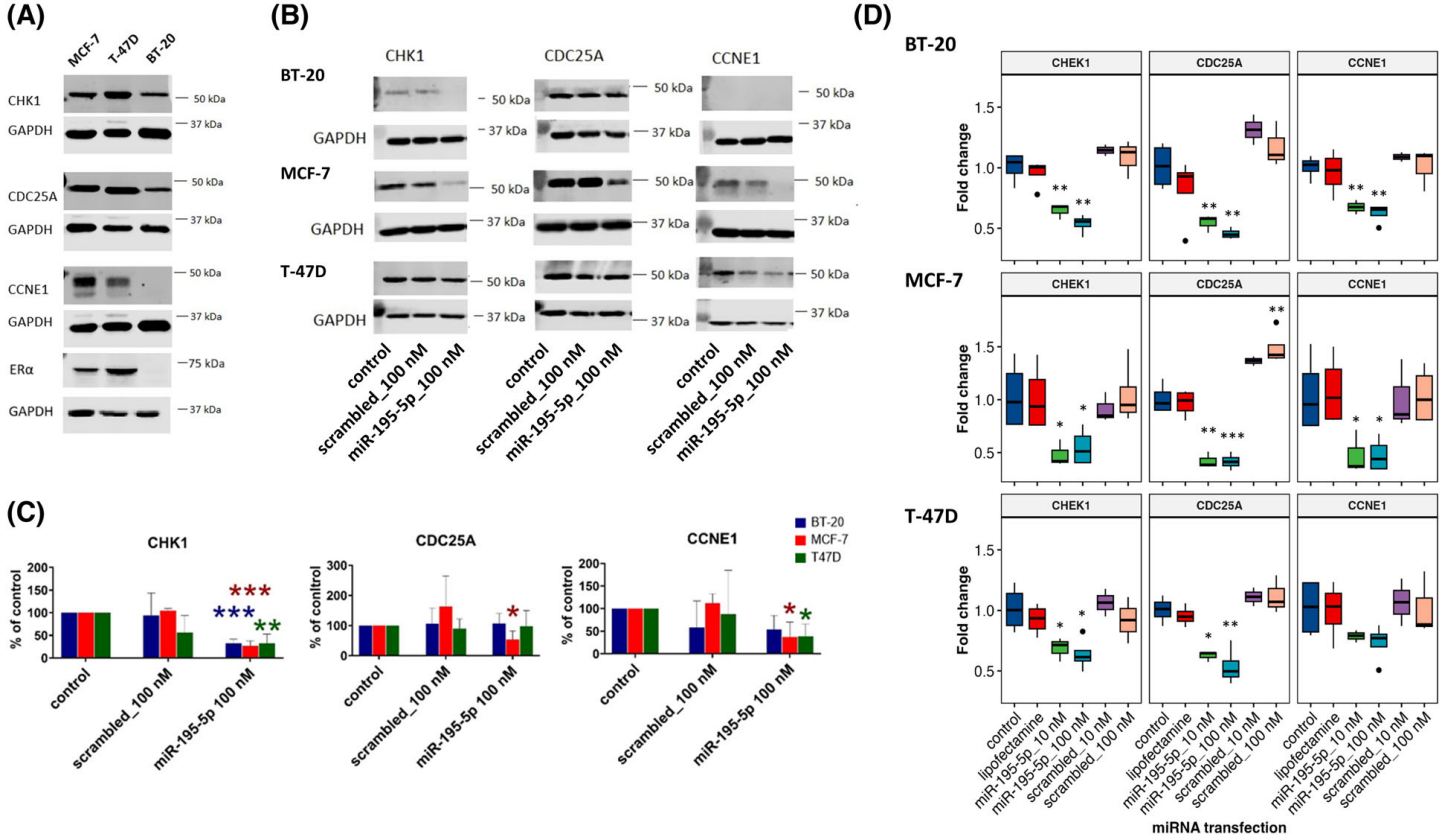
Relative changes of the CHEK1, CDC25A, and CCNE1 gene expression in breast cancer cell lines transfected with miR‐195‐5p miRNA mimics compared to control *in vitro*. Baseline protein expression of the CHEK1, CDC25A, CCNE1, and ERα across BT‐20, MCF‐7, and T‐47D breast cancer cell lines is shown in (A). Relative changes in protein (B, C) and mRNA levels (D) in the same cell lines transfected with miR‐195‐5p miRNA mimics or negative controls for 24 h are presented. Cell lines treated with Lipofectamine RNAiMAX alone or with scrambled miRNAs were used as negative controls. The genes were considered significantly differentially expressed at the transcript level if the absolute value of fold change compared to control was ≥1.5 and *P*‐value ≤0.05. The upper and lower lines of the boxplots represent the upper (75%) and lower quartile (25%), the line across the box represents the median, and the solid circles indicate outliers. Results of transcript expression measured by qPCR are presented as the mean ± SD of at least three independent experiments. Densitometric data of protein expression measured by western blot are presented as a percentage of the control (mean ± SD of three independent experiments), except for CDC25A in T‐47D cells and CCNE1 in BT‐20 cells (based on two replicates due to lack of expression in the third experiment). Western blot images show a representative experiment. Data were analyzed with an unpaired Student's *t*‐test; *P < 0.05, **P < 0.01, and ***P < 0.001.

**Fig. 5:**
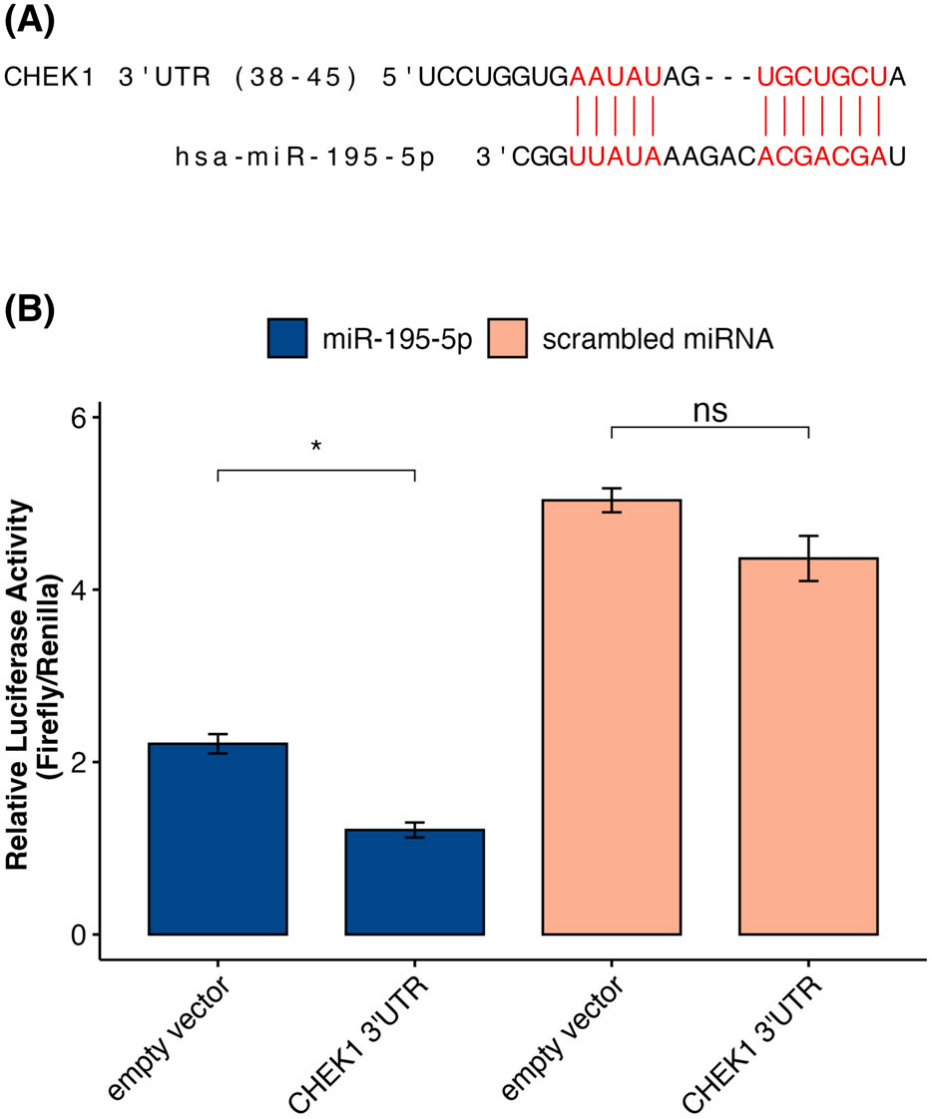
Schematic representation of the binding site between miR‐195‐5p and CHEK1 3′UTR (A) and dual‐luciferase reporter assay demonstrating the regulatory interaction between miR‐195‐5p and CHEK1 (B). MCF‐7 cells were sequentially transfected with a miR‐195‐5p mimic, followed by a reporter vector containing the 3′UTR region of CHEK1 or an empty vector as a control. Luciferase activity was measured 24 h after the second transfection (*P < 0.05, ns, not significant, Student's *t*‐test). Data are presented as mean ± SD from three independent experiments.

The opposite prognostic roles of miR‐195‐5p expression compared to CHEK1 and CDC25A gene expression in luminal BC patients suggested the functional relevance of this miRNA for CHEK1 and CDC25A in this cancer subtype. We thus attempted to verify these interactions using the *in vitro* transfection of miRNA mimics into two ER+ (MCF‐7, T‐47D) and one ER‐ (BT‐20) breast cancer cell lines. First, we determined the baseline expression levels of CHEK1, CDC25A, and CCNE1 in these three cell lines. The largest differences among the lines were in the mRNA and protein CHEK1 expression. It was significantly higher in the T‐47D cell line in comparison to the other lines (Fig. S4). Moreover, its expression clearly correlated with ER protein levels (Fig. 4A). The differences in expression of the other two proteins were negligible at the mRNA level and did not correlate with protein expression; for example, CCNE1 expression was not detectable at the protein level in the BT‐20 cell line. Next, our data showed that overexpression of miR‐195‐5p using miRNA mimics significantly decreased (fold change ≥1.5 and *P* ≤ 0.5) mRNA levels of CHEK1 in all three cell lines, which was also confirmed at the protein level (Fig. 4B–D; Table 14). The effect of miR‐195‐5p mimics treatment after 48 h at the mRNA level and after treatment with lower concentrations of miRNA mimics at the protein level is shown in Figs S5 and S6. The regulatory interaction between miR‐195‐5p and CHEK1 3′UTR was subsequently confirmed using a luciferase reporter assay (Fig. 5).

**Fig. 6:**
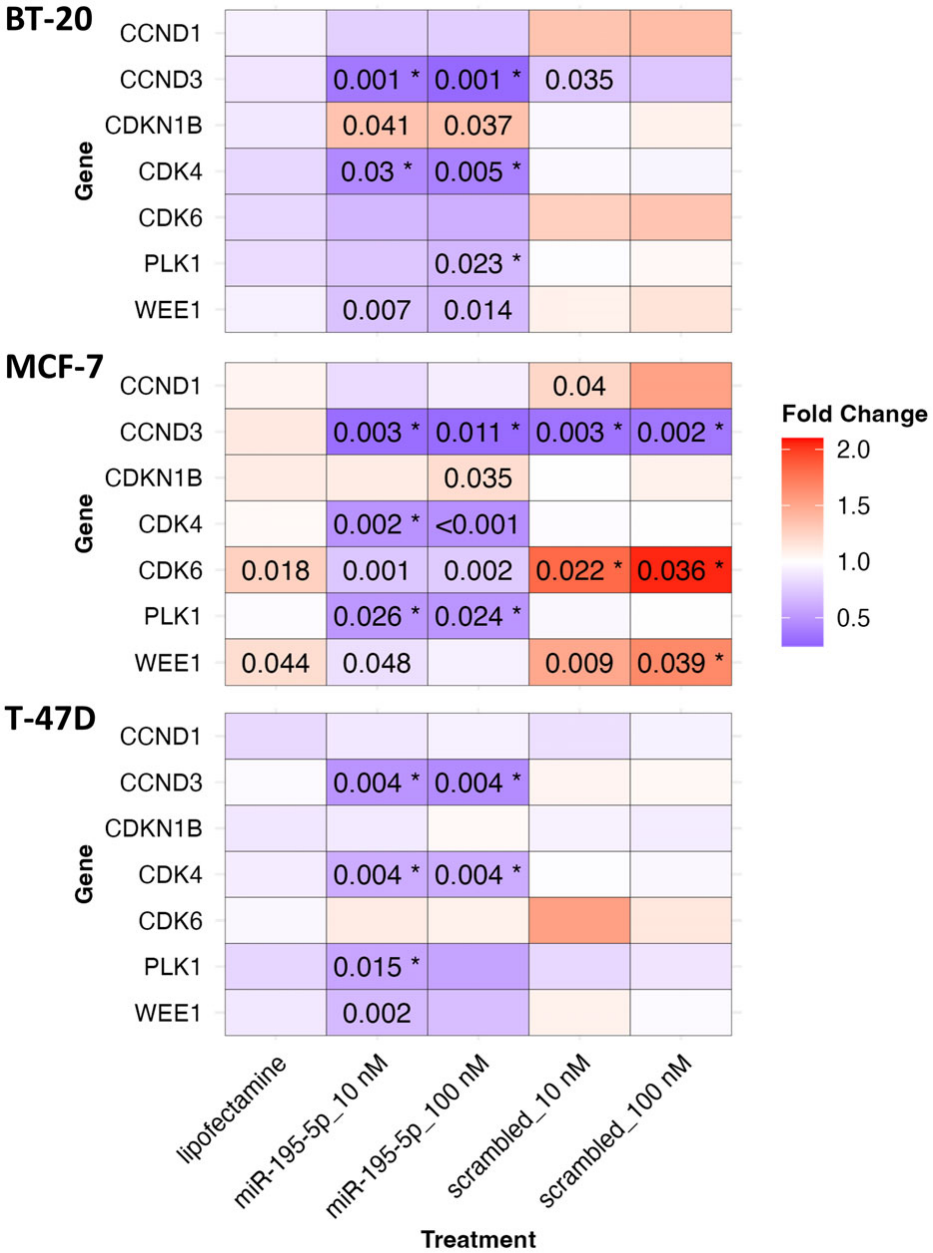
Relative expression changes in the expression of selected CHEK1‐associated genes in breast cancer cell lines transfected with miR‐195‐5p miRNA mimics compared to control *in vitro*. Relative changes in mRNA levels of CCND1, CCND2, CCND3, CDKN1B, CDK4, CDK6, PLK1, and WEE1 in BT‐20, MCF‐7, and T‐47D breast cancer cell lines transfected with miR‐195‐5p miRNA mimics for 24 h, measured by qPCR. Cell lines treated with Lipofectamine RNAiMAX alone or with scrambled miRNAs were used as negative controls. The genes were considered differentially expressed and marked with * if the absolute value of fold change compared to control was ≥1.5 and *P*‐value ≤0.05 (Student's *t*‐test). Presented data are derived from three independent experiments. The expression levels of CCND2 were below the detection limit of the quantification method.

Since miR‐195‐5p has been predicted to regulate additional genes involved in CHEK1‐mediated cell cycle control and DNA damage response, we analyzed the expression of its predicted targets, including CCND1, CCND2, CCND3, CDKN1B, CDK4, CDK6, and WEE1. Additionally, we included PLK1 as another theoretically relevant gene. The overexpression of miR‐195‐5p using miRNA mimics resulted in a significant decrease in mRNA levels of CCND3, CDK4, and PLK1 in all three studied cell lines (Fig. 6).

### 3.5 Rabusertib potentiates doxorubicin cytotoxicity in contrast with miR‐195‐5p *in vitro*

**Fig. 7:**
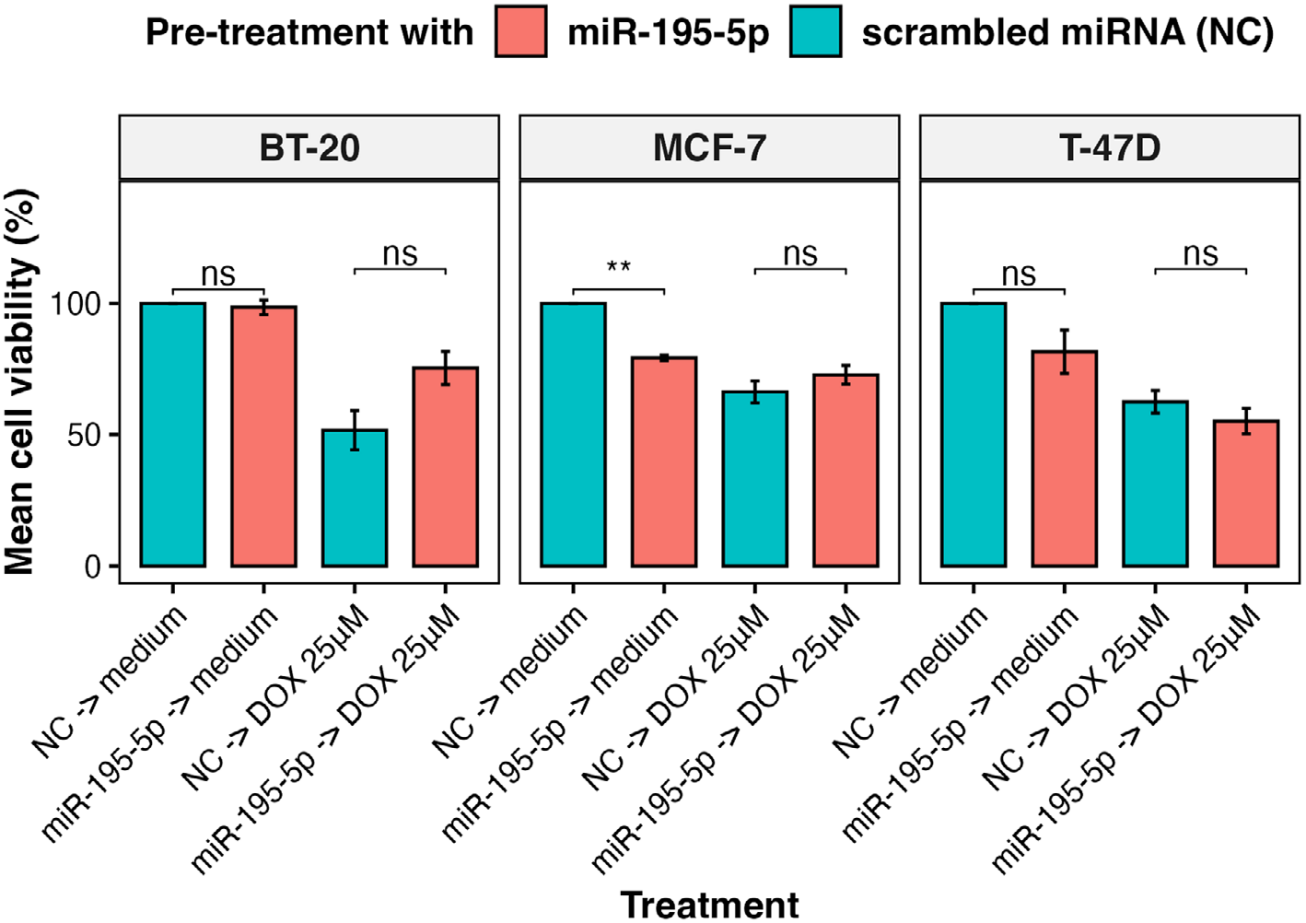
Cell viability of breast cancer cell lines treated with miR‐195‐5p mimics and doxorubicin *in vitro*. DOX = 25 μm doxorubicin, NC = negative control. Cell viability was measured using the CellTiter‐Blue Cell Viability Assay. Data were analyzed with unpaired Student's *t*‐test and presented as mean ± SD from three independent experiments; **P < 0.01, ns, not significant.

Considering the above prognostic significance of CHEK1 and miR‐195‐5p for chemotherapy‐treated patients and verified miRNA‐mRNA regulatory interactions, we hypothesized that the downregulation of CHEK1 by miR‐195‐5p could increase the efficacy of anthracycline doxorubicin in luminal BC cell lines. Thus, we pre‐treated all three BC cell lines with 100 nm miR‐195‐5p miRNA mimics for 24 h and subsequently incubated cells for the next 24 h with doxorubicin (25 μm, according to the previously obtained IC_25_ in parent cell lines, Table 2). As illustrated in Fig. 7, the pretreatment with miR‐195‐5p did not change the doxorubicin efficacy in any of the cell lines. The treatment with miR‐195‐5p alone significantly reduced cell viability only in the MCF‐7 cell line. However, no evidence of apoptosis induction was observed in these cells (Fig. S7).

**Table 2:** The IC_50_ and IC_25_ values obtained for doxorubicin and rabusertib after 24 h treatment of parent breast cancer cell lines *in vitro*. All values in μm, ER = estrogen receptor. IC_50_ and IC_25_ represent the concentrations of the tested compound required to inhibit 50% and 25% of the biological activity, respectively.

Cell line	ER	Doxorubicin		Rabusertib	
		IC_50_	IC_25_	IC_50_	IC_25_
BT‐20	−	45.1 ± 4.0	27.4 ± 2.5	35.0 ± 14.6	7.3 ± 2.4
MCF‐7	+	38.6 ± 14.9	11.1 ± 3.7	20.5 ± 4.9	16.8 ± 4.9
T‐47D	+	68.8 ± 7.4	22.0 ± 2.4	16.6 ± 2.9	12.3 ± 1.3

**Fig. 8:**
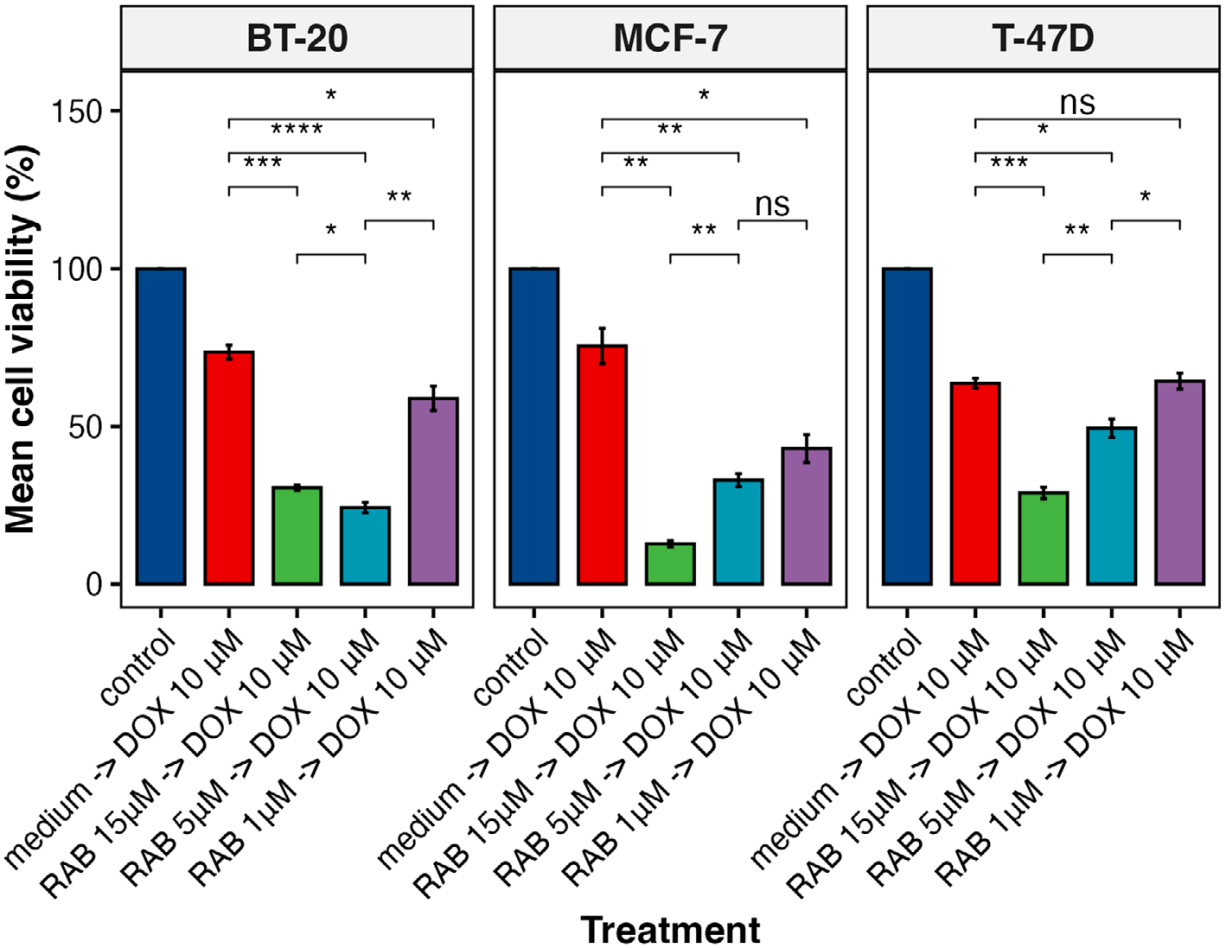
Cell viability of breast cancer cell lines treated with doxorubicin or rabusertib *in vitro*. DOX = 10 μm doxorubicin, RAB = 1–15 μm rabusertib. Cell viability was measured using the CellTiter‐Blue Cell Viability Assay. Data were analyzed with an unpaired Student's *t*‐test and presented as mean ± SD from three independent experiments; *P < 0.05, **P < 0.01, ***P < 0.001, ****P < 0.0001, ns, not significant.

To further investigate the potential of Chk1 inhibitors on doxorubicin‐induced cytotoxicity, we pre‐treated cells with the Chk1‐specific inhibitor rabusertib (LY2603618) at 1, 10, and 15 μm concentrations (chosen based on the highest IC_25_ in the studied cell lines (Table 2)) and subsequently treated the cells with 10 (Fig. 8) or 25 μm (Fig. S8) doxorubicin. Rabusertib showed the highest overall effect on the cell viability at 15 μm concentration in all three cell lines regardless of doxorubicin concentration. However, for the lowest used rabusertib concentration (1 μm), we have observed the highest cell viability reduction in the ER+ MCF‐7 cell line when subsequently treated with 10 μm of doxorubicin (Fig. 8).

## 4 Discussion

In the present study, the high expression levels of CHEK1 and CDC25A and low expression levels of hsa‐miR‐195‐5p were associated with poor RFS in luminal BC patients treated with chemotherapy. Because of this opposite clinical significance and the predicted miRNA‐mRNA interactions, we used transfection of hsa‐miR‐195‐5p miRNA mimics to one ER‐ and two ER+ breast cancer cell lines and identified hsa‐miR‐195‐5p as a negative regulator of CHEK1 independently of ER expression.

Hsa‐miR‐195 is co‐expressed within the miR‐497∼195 cluster along with hsa‐miR‐497 encoded by the miRNA host gene MIR497HG on human chromosome 17p13.1 (Gene ID: 100506755, alternative names: ENSG00000267532, lnc‐SLC16A11–7). The miR‐195‐5p and miR‐497‐5p or the MIR497HG lncRNA exhibit a general downregulation pattern in BC compared to adjacent mammary tissue, which we have confirmed in our study. This downregulation is similar across various tumor subtypes and these microRNAs are recognized as tumor suppressors [29, 30, 31], an assumption which is in agreement with our results, that is, poor RFS in patients with low expression of miR‐195‐5p in their tumors. Both miRNAs also clustered together with hsa‐let‐7c‐5p, hsa‐miR‐99a‐5p, hsa‐miR‐100‐5p, hsa‐miR‐125b‐5p, hsa‐miR‐143‐3p, hsa‐miR‐145‐5p, hsa‐miR‐199a‐3p, hsa‐miR‐199a‐5p, hsa‐miR‐199b‐5p, and hsa‐miR‐214‐3p among miRNAs with relatively high expression in breast carcinoma tissues, suggesting their potentially greater biological relevance.

CHEK1, CDC25A, and CCNE1, together with CCND1, CCND3, CDK4, CDK6, BTRC, E2F3, and FOXK1, were previously identified as the targets of miR‐497∼195 cluster in hepatocellular carcinoma [32, 33]. Independently, miR‐195‐5p has been described as a regulator of the CHEK1 gene in non‐small cell lung and gastric carcinoma [34, 35], and CCNE1 in glioma [36]. As regards BC, so far, only CCNE1 was identified as the target of hsa‐miR‐195‐5p in the triple‐negative subtype [37, 38], and thus, our observation for luminal subtypes is novel.

CHEK1 (Chk1) is a serine/threonine kinase encoded by the CHEK1 gene and the master regulator of DNA damage response (DDR). In the case of replication stress, CHEK1 is activated by ATR serine/threonine kinase and induces cell cycle arrest at the intra‐S checkpoint through the phosphorylation and subsequent degradation of phosphatase CDC25A, thereby inhibiting the activity of CDK2 and CCNE1 [39].

Classical chemotherapeutics as topoisomerase inhibitors (e.g., anthracyclines or etoposide) exert cytotoxic effects mainly through the induction of DNA damage, which activates the ATR/CHEK1 pathway [40, 41]. Considering the demonstrated significance of CHEK1 and miR‐195‐5p for the prognosis of patients treated with classical chemotherapy and their verified miRNA‐mRNA regulatory relation in the present study, we hypothesized that the downregulation of CHEK1 by miR‐195‐5p could increase the efficacy of anthracyclines in luminal BC. However, the inhibition of the CHEK1‐CDC25A‐CCNE1 axis with the hsa‐miR‐195‐5p did not affect the efficacy of doxorubicin in our *in vitro* experiments, while the specific CHEK1 chemical inhibitor rabusertib was considerably potent. Among the potential reasons for this observation, we can list the existence of other CHEK1 miRNA regulators predicted in our study, apart from hsa‐miR‐195‐5p, such as hsa‐miR‐497‐5p, hsa‐miR‐199a/b‐5p, or hsa‐miR‐100‐5p. These miRNAs showed a strong correlation with each other and a negative correlation with CHEK1, CDC25A, and CCNE1, suggesting their involvement in the regulation of the CHEK1‐CDC25A‐CCNE1 axis. However, only hsa‐miR‐195‐5p and hsa‐miR‐497‐5p were predicted for all three genes, suggesting they have the strongest inhibitory and functional effect. Also, other additional members of the miR‐15 family were confirmed to regulate CHEK1 in BC (hsa‐miR‐15a, hsa‐miR‐15b, and hsa‐miR‐16) [42, 43] or other carcinomas (hsa‐miR‐424) [44]. Nevertheless, among this miRNA precursor family, only hsa‐miR‐195‐5p and hsa‐miR‐497‐5p were associated with clinical data of luminal BC patients and, in the case of hsa‐miR‐195‐5p, with their prognosis in our study, indicating their specific importance within this BC subtype.

Vice versa, several predicted or confirmed mRNA targets of hsa‐miR‐195‐5p have been well described. Our study further revealed that miR‐195‐5p transfection led to the downregulation of CCND3, CDK4, and PLK1 in all studied breast cancer cell lines. These findings suggest that miR‐195‐5p has a broader effect on the cell cycle and DDR, potentially playing some role in regulating the G1 to S phase transition (CCND3, CDK4) [45], which may lead to cell cycle arrest in the G1 phase, where doxorubicin is less effective. In contrast, the direct inhibition of CHEK1 by rabusertib primarily blocks the G2/M checkpoint leading to replication stress, causing cells exposed to doxorubicin to proceed into mitosis with damaged DNA, ultimately resulting in irreversible mitotic catastrophe and cell death [46]. This combination could also be effective in other types of tumors. A recent study demonstrated that inhibition of the ATR‐CHK1 pathway synergizes with doxorubicin in inducing cytotoxicity in acute lymphoblastic leukemia cells [41]. However, no clinical trial has been conducted or is currently ongoing that combines these two drugs. A phase II study (NCT00839332) tested rabusertib with gemcitabine in pancreatic carcinoma, but it showed no advantage over gemcitabine alone [47]. Another phase II trial (NCT01139775) assessed rabusertib with pemetrexed and cisplatin in advanced nonsquamous non‐small cell lung cancer (NSCLC), but development was discontinued due to a potential increased risk of thromboembolic events. Future research should also investigate other CHK1 inhibitors, such as prexasertib, which remains the most advanced CHK1 inhibitor in clinical development [48].

Since ER+ patients typically undergo hormonal therapy, regardless of the use of chemotherapy or anti‐HER2 therapy, it remains plausible that the low levels of hsa‐miR‐195‐5p may be linked to resistance against hormonal treatment. Tian et al. recently showed that reduced expression of miR‐497/195 or MIR497HG can promote estrogen‐independent proliferation and tamoxifen resistance *in vitro* with the MCF‐7 cell line and *in vivo* using a cell‐derived xenograft model [49]. Moreover, according to our results, SMAD3 and SMAD7 are predicted targets of miR‐195‐5p, and their altered expression is associated with epithelial‐to‐mesenchymal transition (EMT) in breast carcinoma, as reported in previous studies [28, 50]. This effect may be particularly significant in luminal breast carcinoma patients, given the functional relationship between SMAD3/SMAD4, TGF‐β, and ERα [51]. In combination with low miR‐195‐5p levels, this could contribute to enhanced EMT and resistance to endocrine therapy in ER+ patients. Thus, the role of hsa‐miR‐195‐5p and hsa‐miR‐497‐5p in hormonal therapy and EMT of luminal BC has yet to be verified in a study with a sufficient number of patients. The present study was not designed to provide such information.

In our study, low CCNE1 expression was associated with shorter RFS in patients treated with adjuvant chemotherapy. We assume that the contrasting impact of CCNE1 on RFS, compared to the role of CHEK1, may be subtype‐specific. Our results show that high CHEK1 expression was associated with shorter RFS, particularly in patients with the luminal A subtype, whereas the prognostic significance of CCNE1 was observed mainly in luminal B patients. CCNE1 protein expression may be regulated differently among luminal breast carcinoma patients, which corresponds to the challenges associated with correlating CCNE1 protein and mRNA levels [52]. For example, in our cohort, CCNE1was the only gene found to be significantly overexpressed in tumors lacking progesterone receptor (PR) expression. Additionally, other miRNAs that have been reported to directly bind to and regulate CCNE1 expression in breast carcinoma, including hsa‐miR‐15a, hsa‐miR‐483‐3p, and hsa‐miR‐30c‐2‐3p, may also contribute to this differential regulation [53, 54, 55]. However, their clinical significance in the luminal breast carcinoma subtype was not confirmed in our study. Similarly, CCNE1 gene amplification, which is often associated with higher CCNE1 expression and shorter RFS in triple‐negative breast carcinoma, is relatively rare in the luminal subtype [52, 56].

The main limitation of the present study is the limited sample size of a subgroup of patients with luminal A subtype of breast carcinoma and the lack of cell models reliably representing different luminal subtypes [57]. Nevertheless, comprehensive *in silico* prediction, followed by prioritization focused on breast carcinoma‐relevant targets, has identified a potential candidate for further *in vivo* studies, which are beyond scope of the current study.

## 5 Conclusions

In conclusion, we identified a specific miRNA signature associated with highly proliferative luminal breast carcinomas, the ability of hsa‐miR‐195‐5p to inhibit CHEK1 expression in breast carcinoma *in vitro*, and the promising potential for pretreatment of a subset of patients with luminal tumors with Chk1 inhibitors to enhance the efficacy of chemotherapy, underscoring the importance of CHEK1 expression in the prognosis and treatment of patients with luminal breast carcinomas.

## Conflict of interest

The authors declare no conflict of interests.

## Author contributions

VB and PS: conceptualization; VB: methodology; VB, ME, AS, IK, SS, VH, and VN: investigation; VB and PS: formal analysis; RK, MT, DV, JG, KK, MM, and SM: data curation; VB: writing—original draft; VB, PS, AS, VN, VH, IK, and MM: writing—review & editing; PS and VH: funding acquisition; VB and PS: supervision. All authors read and approved the final version of the manuscript.

## Peer review

The peer review history for this article is available at https://www.webofscience.com/api/gateway/wos/peer‐review/10.1002/1878‐0261.70077.

## Supporting information


**Fig. S1.** Clusters proposed by MCL (Markov Cluster Algorithm) cluster analysis (STRING) composed of two or more proteins, including 84 from the originally input 200 genes.
**Fig. S2.** Correlation matrix for 19 genes selected based on the cluster analysis and literature review from the 200 potential target genes.
**Fig. S3.** Overall survival probabilities for breast carcinoma patients stratified by subtype, therapy and expression of studied genes.
**Fig. S4.** Relative CHEK1, CDC25A, and CCNE1 gene expression in parental breast cancer cell lines *in vitro*.
**Fig. S5.** Relative changes of the CHEK1, CDC25A, and CCNE1 gene expression in breast cancer cell lines transfected with miR‐195‐5p miRNA mimics compared to control *in vitro* after 24 h and 48 h.
**Fig. S6.** Protein expression of CHK1, CDC25A, and CCNE1 in breast cancer cell lines following miR‐195‐5p treatment.
**Fig. S7.** Western blot analysis of cleaved caspase‐3 (a) and PARP (b) protein expression in breast cancer cell lines following miR‐195‐5p treatment.
**Fig. S8.** Cell viability of breast cancer cell lines treated sequentially with rabusertib and doxorubicin.

**Table S1.** Clinical characteristics of luminal breast carcinoma patients used for miRNome profiling (*N* = 101).
**Table S2.** The list of TaqMan Gene and miRNA expression assays.
**Table S3.** Quantile normalized and log2 transformed expression levels of 2006 miRNA determined in luminal breast carcinoma samples (*N* = 101).
**Table S4.** Significantly dysregulated miRNAs in luminal breast carcinomas stratified by grade.
**Table S5.** Significantly dysregulated miRNAs in luminal breast carcinomas stratified by Ki‐67 expression status.
**Table S6.** Significantly dysregulated miRNAs in luminal breast carcinomas stratified by HER2 status.
**Table S7.** Significantly dysregulated miRNAs in luminal B versus luminal A breast carcinomas.
**Table S8.** The list of potential target genes of 25 miRNAs associated with grade and Ki‐67 expression in luminal breast carcinomas.
**Table S9.** Significantly enriched pathways (*N* = 109) revealed by the KEGG pathway's enrichment analysis predicted targets of 25 miRNAs associated with grade and Ki‐67 expression in luminal breast carcinomas.
**Table S10.** The list of miRNA target genes (Table S9) prioritized by help of the DAVID database.
**Table S11.** Associations of relative transcript levels of the genes positively correlated with candidate miRNAs in tumors with clinical data of subgroup of luminal breast carcinoma patients treated with adjuvant chemotherapy (*N* = 79).
**Table S12.** Differences in relative transcript levels of the examined genes between tumors and non‐tumor tissues.
**Table S13.** Associations of relative transcript levels of the examined genes in tumors with clinical data of luminal breast carcinoma patients treated with adjuvant chemotherapy (*N* = 177).
**Table 14.** Densitometry data from western blot analysis of CDC25A, CHK1, and CCNE proteins presented as normalized signals (protein of interest signal divided by GAPDH signal) (a) and as a percentage of the control (calculated from the normalized signals) (b).

## Data Availability

Microarray data supporting this study are available in the Gene Expression Omnibus database (GEO, GSE267543). All other data are available on reasoned request from the corresponding author.
