# Supplementary material for: Integrative miRNOMe profiling reveals the miR‐195‐5p–CHEK1 axis and its impact on luminal breast cancer outcomes
Source: Mol Oncol. 2025 Jun 23;19(11):3409–26. doi: 10.1002/1878-0261.70077 (PMC12591302; doi:10.1002/1878-0261.70077)

**Integrative miRNOMe profiling reveals the miR-195-5p-CHEK1 axis and its impact on luminal breast cancer outcomes**

Veronika Boušková^1,2*^, Marie Ehrlichová^1,2^, Alžběta Spálenková^1,2^, Ivona Krus^1^, Simona Šůsová^1,2^, Viktor Hlaváč^1,2^, Vlasta Němcová^3^, Renata Koževnikovová^4^, Markéta Trnková^5^, David Vrána^6^, Jiří Gatěk^7^, Kateřina Kopečková^8^, Marcela Mrhalová^9^, Soňa Měšťáková^10^, and Pavel Souček^1,2^

^1^Toxicogenomics Unit, National Institute of Public Health, Prague, Czech Republic

^2^Biomedical Center, Faculty of Medicine in Pilsen, Charles University, Pilsen, Czech Republic

^3^Department of Biochemistry, Cell and Molecular Biology, Third Faculty of Medicine, Charles University, Prague, Czech Republic

^4^MEDICON a.s., Prague, Czech Republic

^5^Unilabs Pathology k.s., Prague, Czech Republic

^6^Comprehensive Cancer Center of Hospital AGEL Novy Jicin, Novy Jicin, Czech Republic

^7^EUC Hospital Zlin and Tomas Bata University in Zlin, Zlin, Czech Republic

^8^Department of Oncology, Second Faculty of Medicine, Charles University and Motol University Hospital, Prague, Czech Republic

^9^Department of Pathology and Molecular Medicine, Second Faculty of Medicine, Charles University and Motol University Hospital, Prague, Czech Republic

^10^Department of Surgery, Second Faculty of Medicine, Charles University and Motol University Hospital, Prague, Czech Republic

Corresponding author: Veronika Boušková, PhD, Toxicogenomics Unit, National Institute of Public Health, Srobarova 49/48, Prague 10, 100 42, Czech Republic, email: [veronika.bouskova@szu.cz](mailto:veronika.bouskova@szu.cz)

ORCID: 0000-0002-0758-4657

**Supplementary Figure S1:** Clusters proposed by MCL (Markov Cluster Algorithm) cluster analysis (STRING) composed of two or more proteins, including 84 from the originally input 200 genes

Clusters consisting of a single protein (NCOR2, PGR, and GSK3B) were maintained in the visualization of interactions. The highlighted genes CHEK1, CDC25A, WEE1, CCNE1, CDKN1B, CDK6, and CCNDs were selected for further analysis.


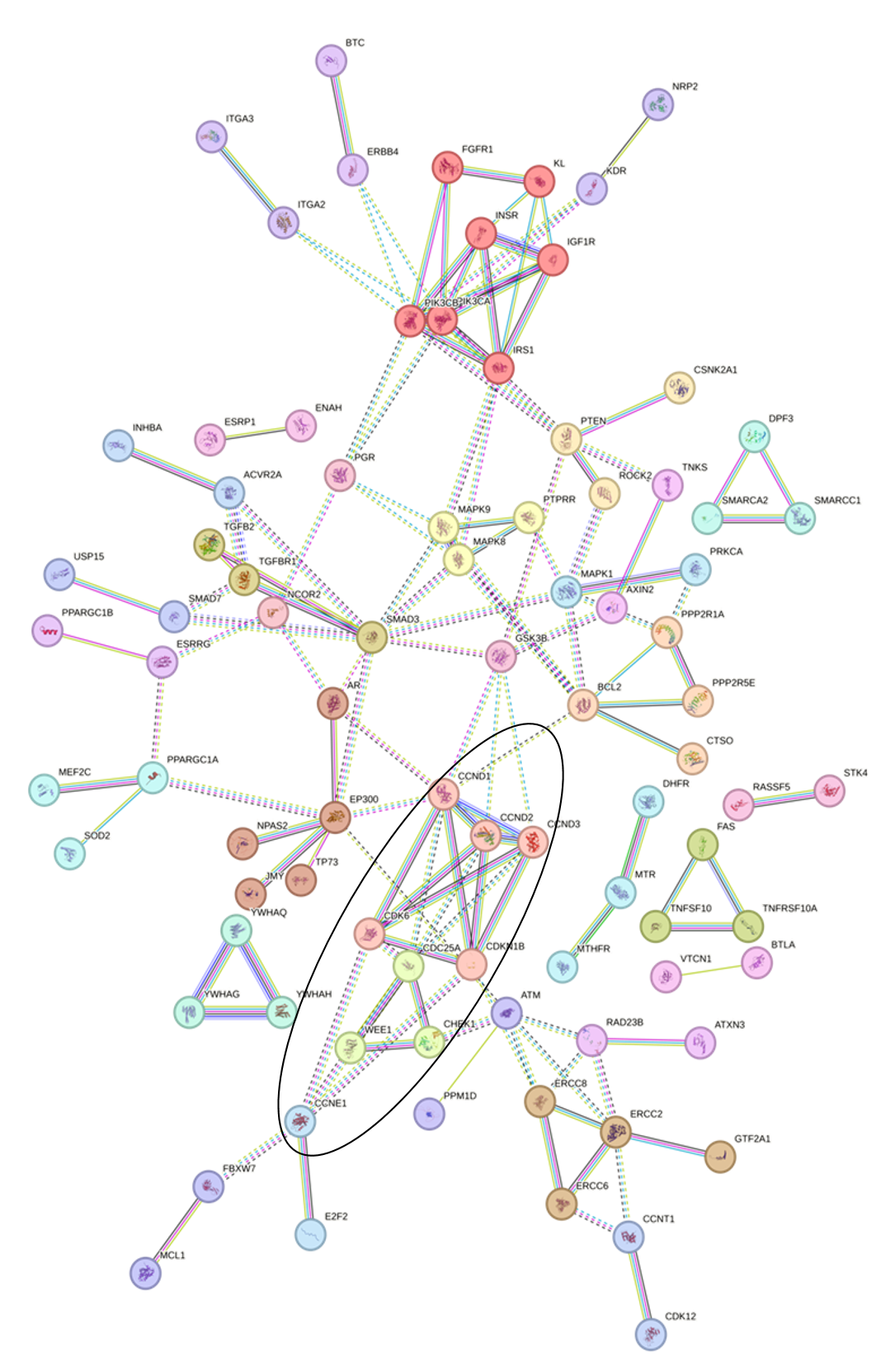


**Supplementary Figure S2:** Correlation matrix for 19 genes selected based on the cluster analysis and literature review from the 200 potential target genes

Genes were clustered using the complete linkage method for better visualization. Blue indicates positive correlations and red indicates negative ones (Spearman 's rank correlation coefficient). The white cells depict statistically insignificant correlations (p ≥ 0.05, Spearman 's rank correlation).

**
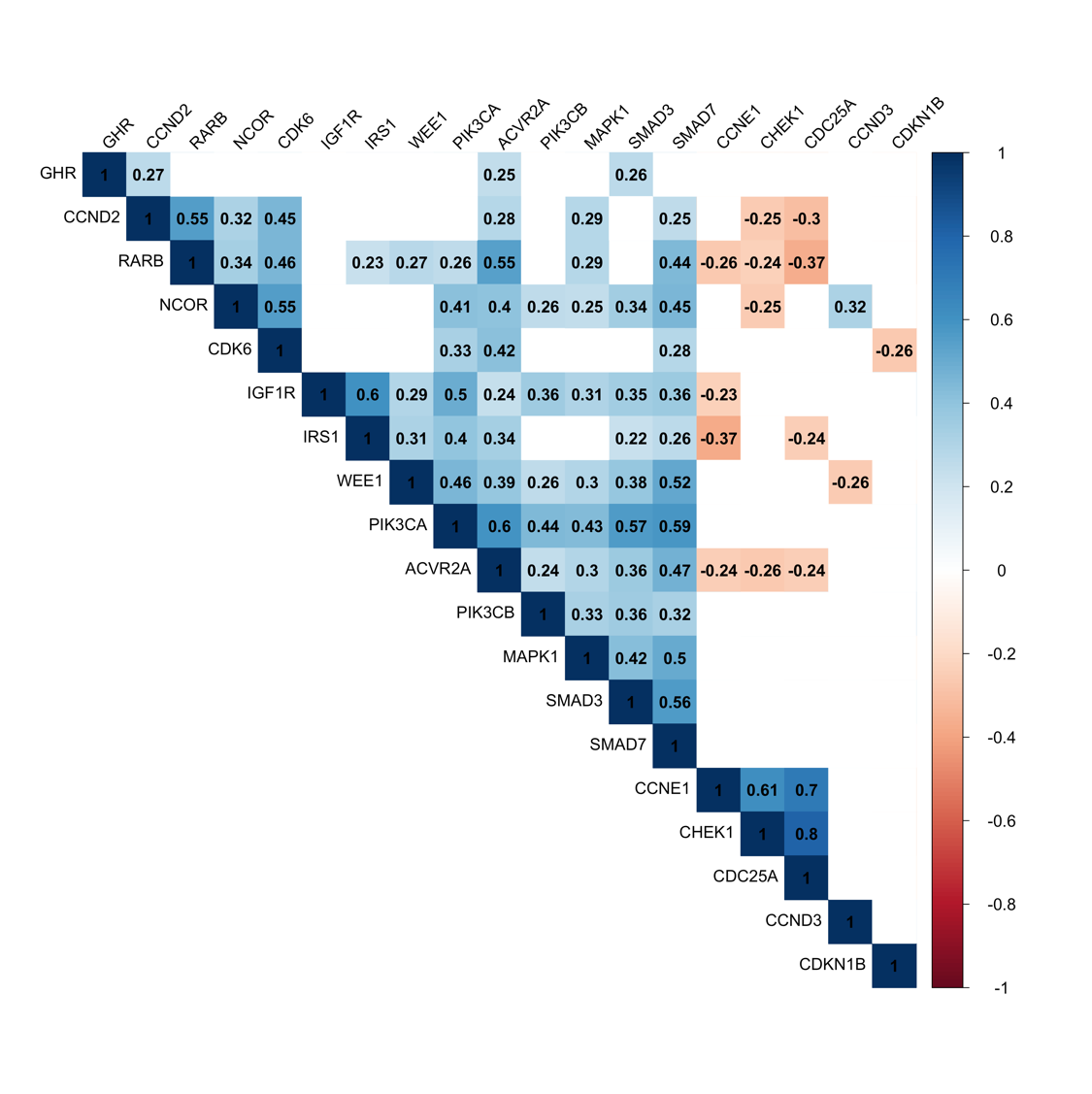
**

**Supplementary Figure S3:** Overall survival probabilities for breast carcinoma patients stratified by subtype, therapy and expression of studied genes

Overall survival of adjuvant chemotherapy treated patients with luminal subtypes (N = 171) (a) or solely the patients with luminal A subtype (N = 54) (**b**) stratified by the CHEK1 gene expression. Overall survival of adjuvant chemotherapy treated patients with luminal subtypes (N = 171) (**c**) and solely the patients with the luminal B subtype (N = 113) (**d**) stratified by the CCNE1 gene expression. Neoadjuvant chemotherapy treated patients with luminal subtypes (N = 38) stratified by miR-195-5p (**e**) and CDC25A (**f**) gene expression levels. The optimal cut-off for dividing patients with low vs high expression was the 50th percentile (Q1-Q2 vs Q3-Q4) unless otherwise specified.

**
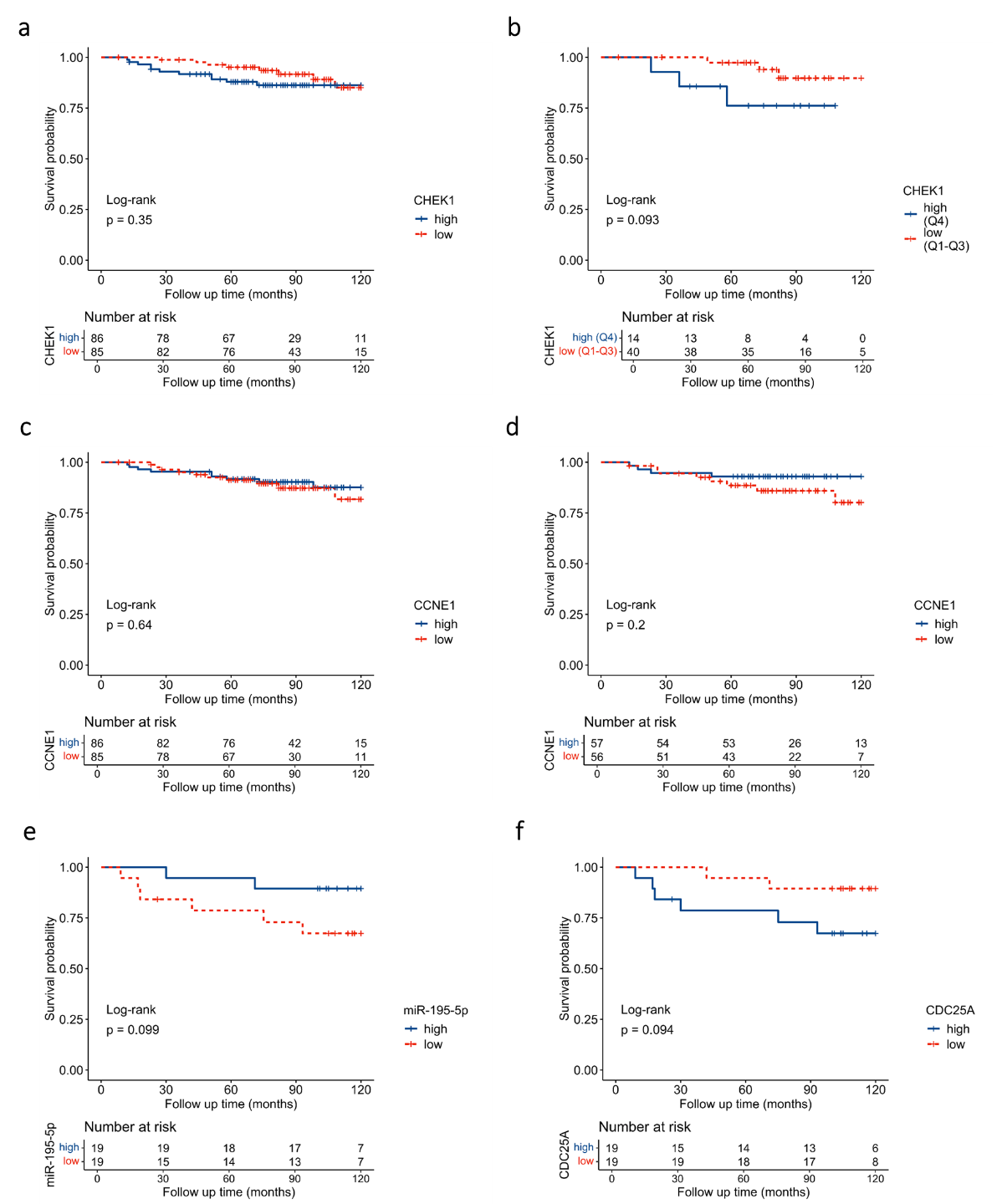
**

**Supplementary Figure S4:** Relative CHEK1, CDC25A, and CCNE1 gene expression in parental breast cancer cell lines *in vitro*

Expression indicated as fold change compared to the sample with the lowest expression (T-47D for CCNE1), measured by qPCR. Data were analyzed with unpaired Student's t-test and presented as mean ± S.D. from two independent experiments (for a basic estimate of baseline expression); *p < 0.05, **p < 0.01, ns = not significant.


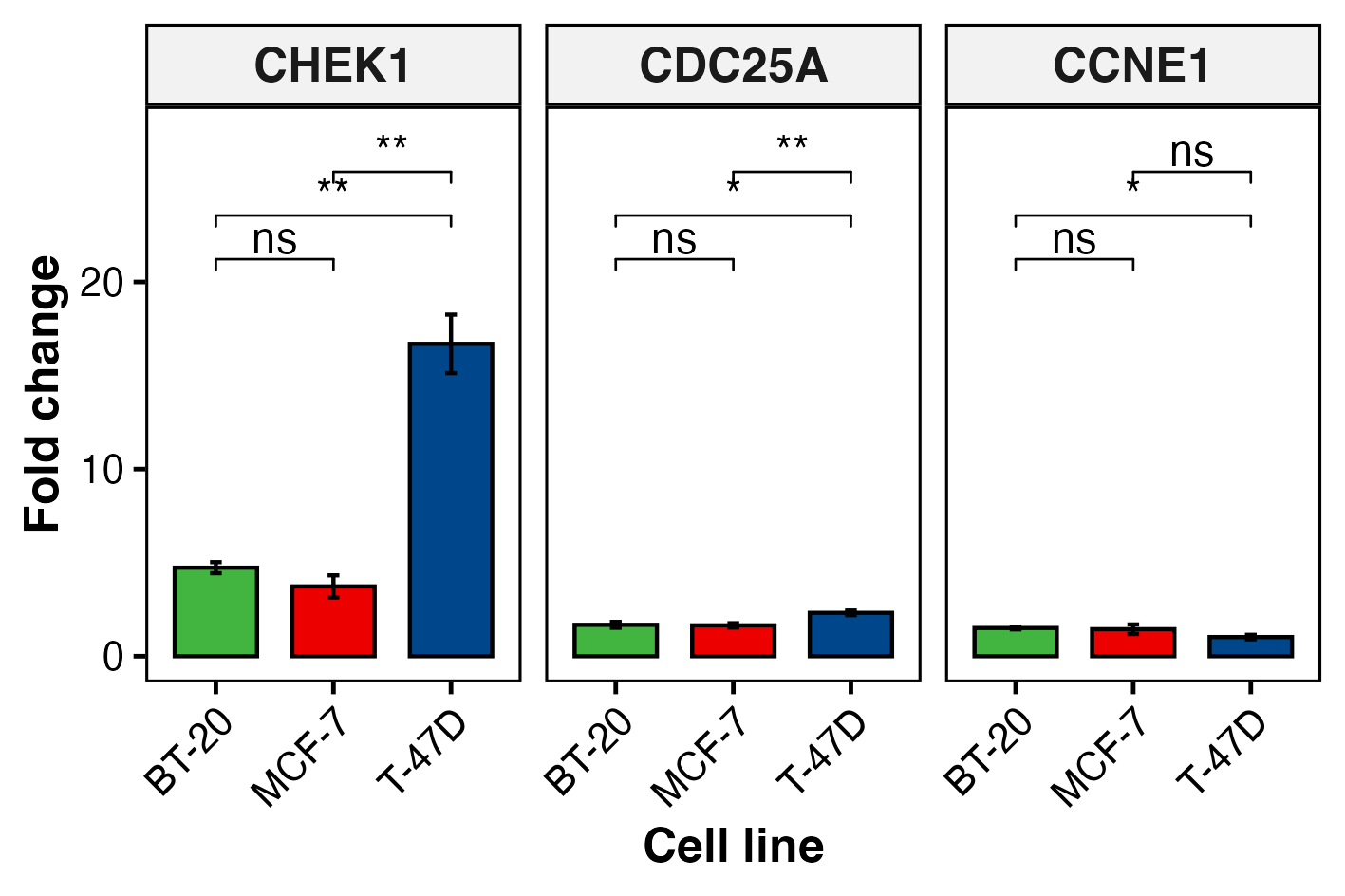


**Supplementary Figure S5:** Relative changes of the CHEK1, CDC25A, and CCNE1 gene expression in breast cancer cell lines transfected with miR-195-5p miRNA mimics compared to control *in vitro* after 24h and 48h

Relative changes in mRNA levels in BT-20, MCF-7, and T-47D breast cancer cell lines transfected with miR-195-5p miRNA mimics or negative controls for 24 h and 48 h. Cell lines treated with Lipofectamine RNAiMAX alone or with scrambled miRNAs were used as negative controls. Presented data are from a single biological experiment and are intended to provide a basic overview of differences in response between the two time points.


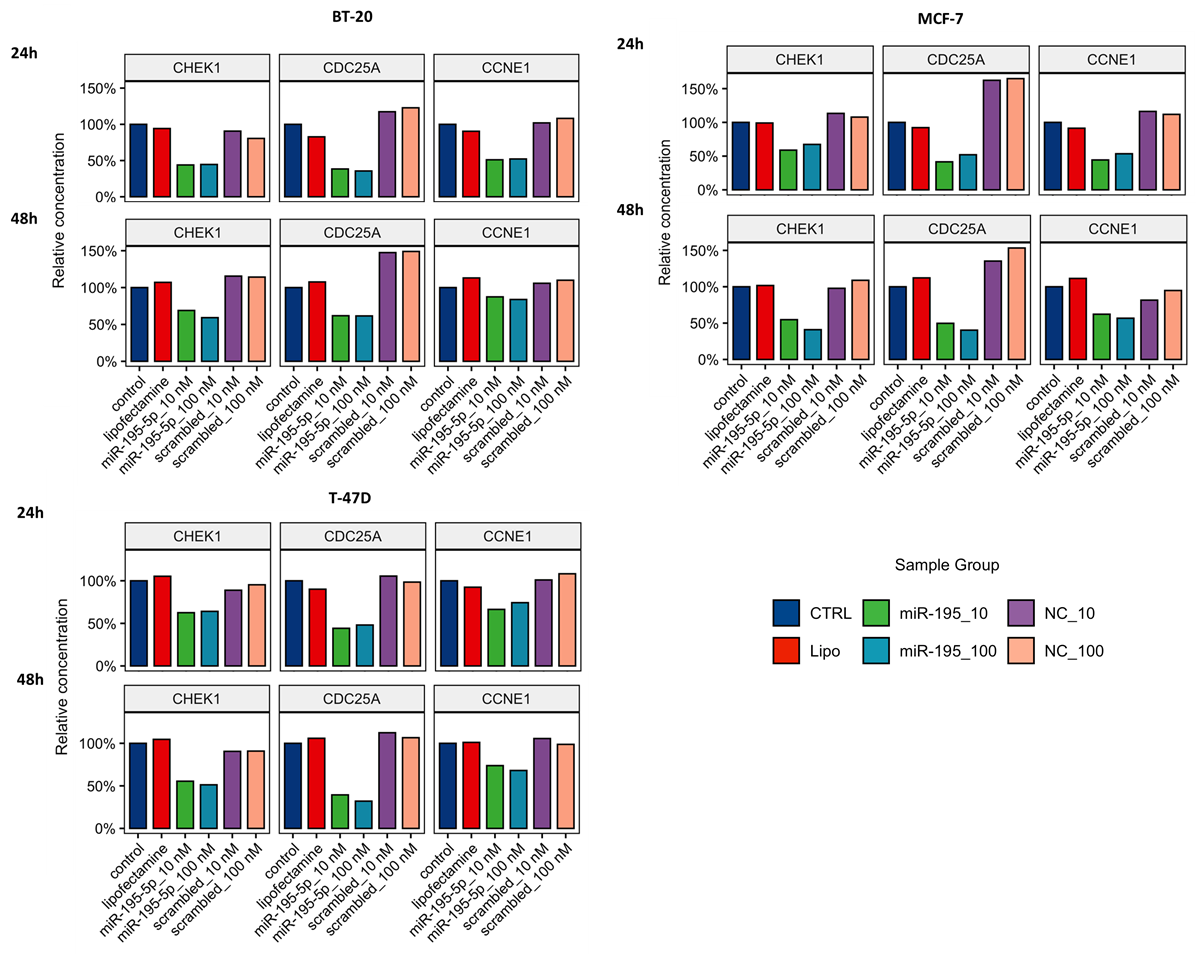


**Supplementary Figure S6:** Protein expression of CHK1, CDC25A, and CCNE1 in breast cancer cell lines following miR-195-5p treatment

Western blot analysis of CHK1 (**a**), CDC25A (**c**), and CCNE1 (**e**) in BT-20, MCF-7, and T-47D breast cancer cell lines transfected with miR-195-5p miRNA mimics or negative (scrambled) controls for 24 h. Densitometric data (**b, d, f**) are shown as a percentage of the control (mean ± S.D. of three independent experiments), except for CDC25A in T-47D cells and CCNE1 in BT-20 cells (based on two replicates due to lack of expression in the third experiment). Data were analyzed with unpaired Student's t-test; *p < 0.05, **p < 0.01, and ***p < 0.001.

**
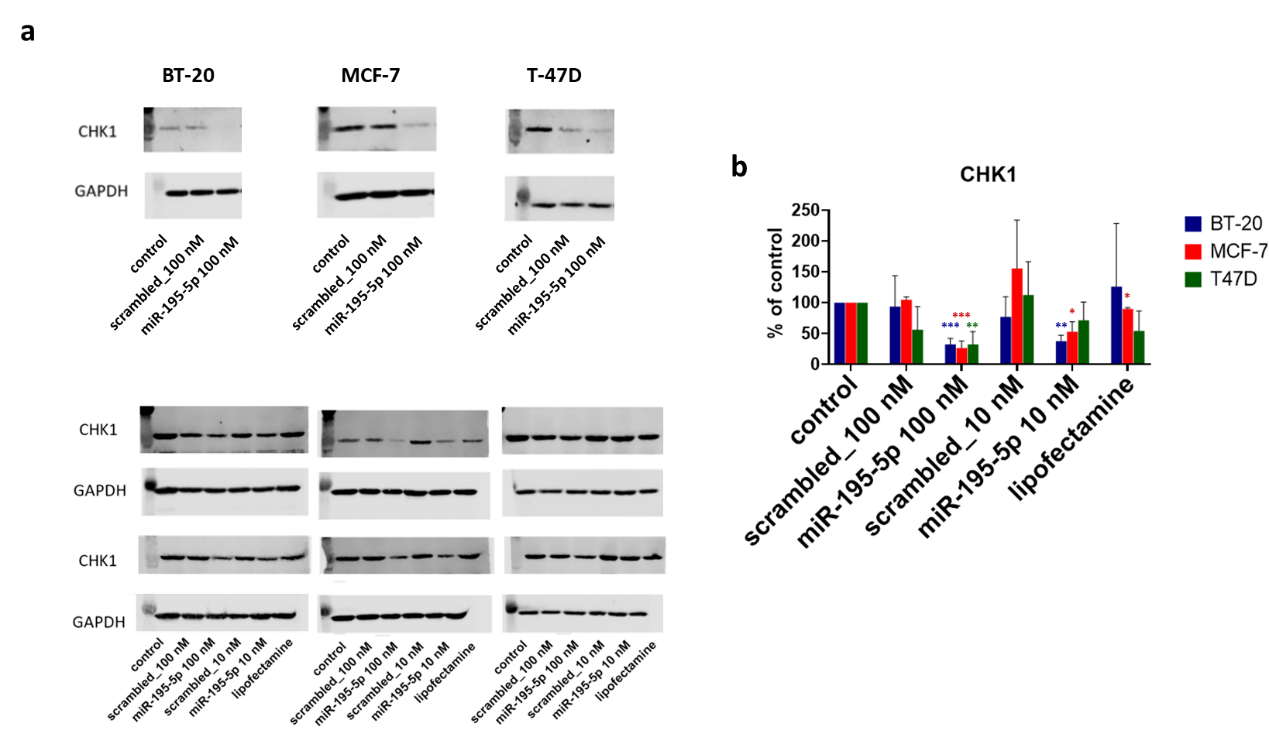
**

**
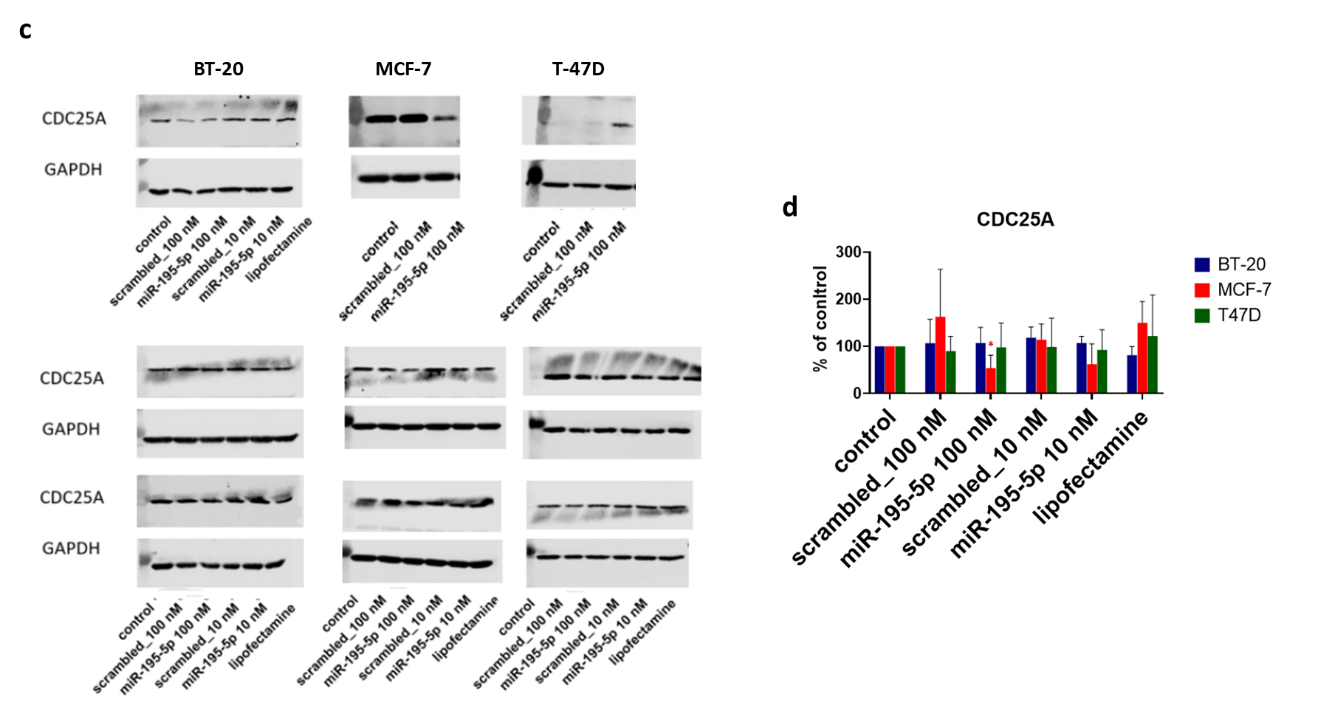
**

**
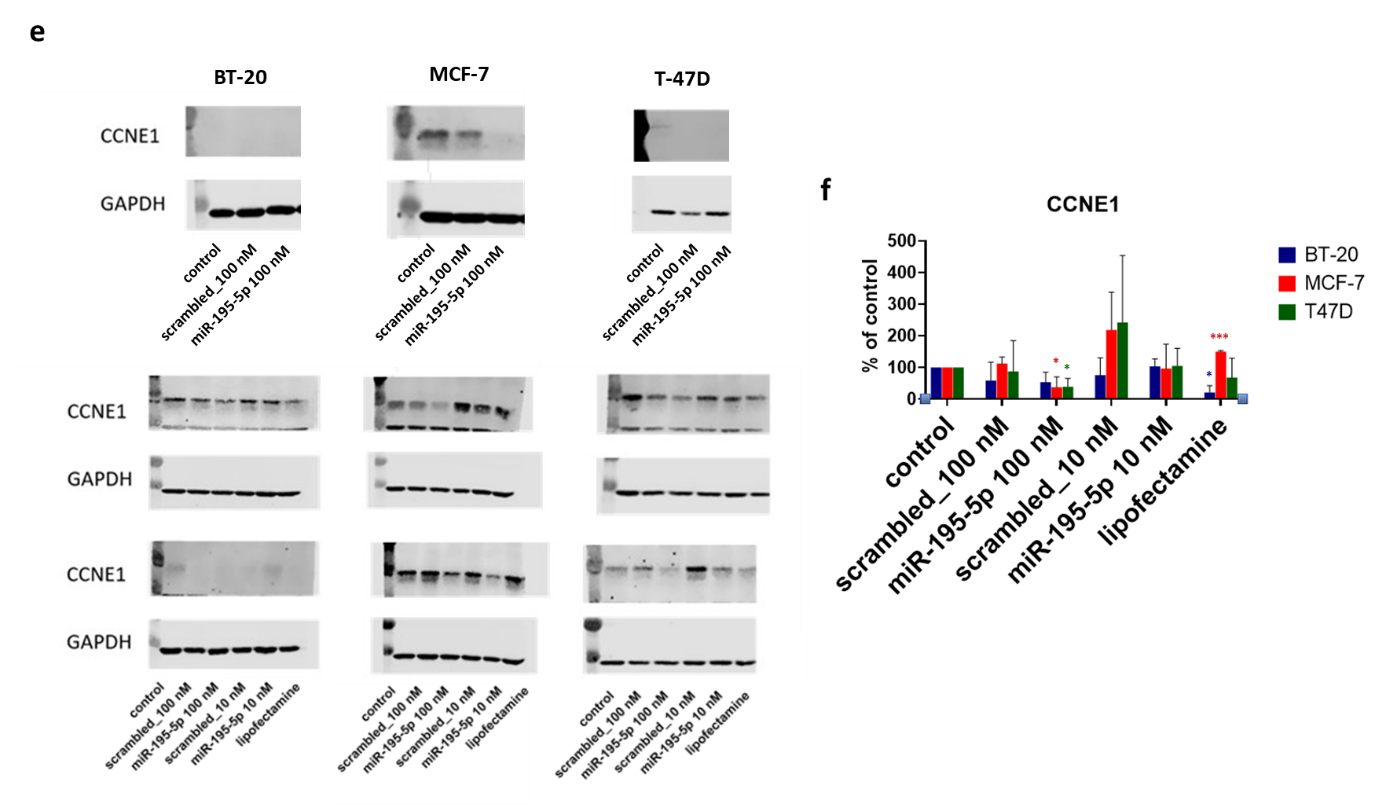
**

**Supplementary Figure S7:** Western blot analysis of cleaved caspase-3 (**a**) and PARP (**b**) protein expression in breast cancer cell lines following miR-195-5p treatment

BT-20, MCF-7, and T-47D breast cancer cell lines were transfected with miR-195-5p miRNA mimics or negative (scrambled) controls for 24 h. Protein extracted from a tumor cell line undergoing apoptosis was used as a positive control. Presented data illustrate a single biological experiment, as no signs of apoptosis were detected.

**
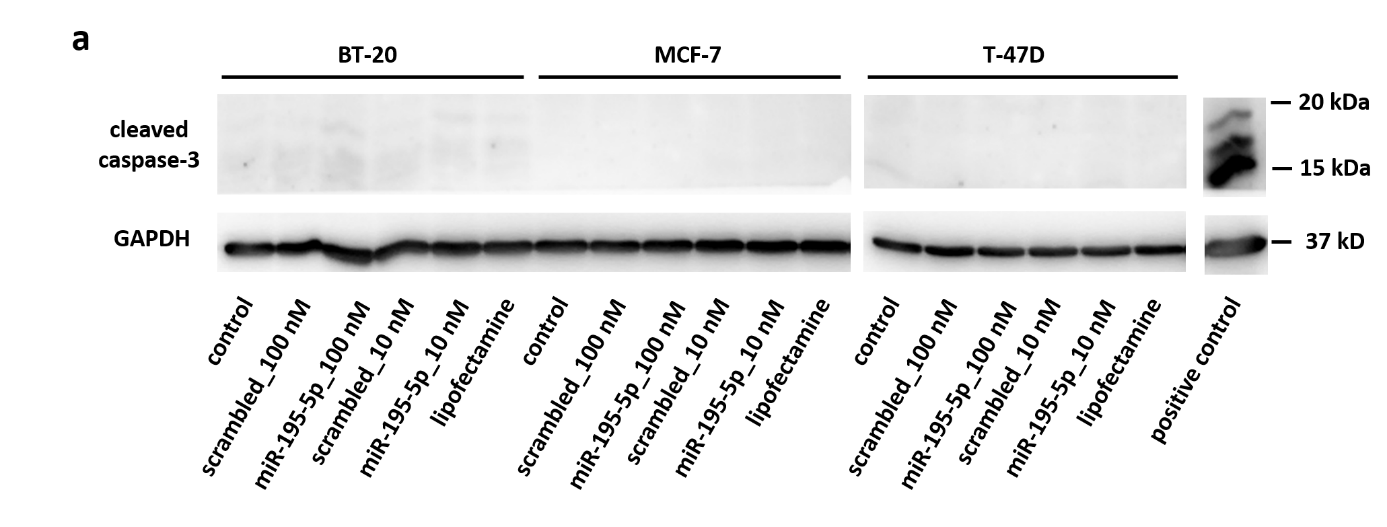
**

**
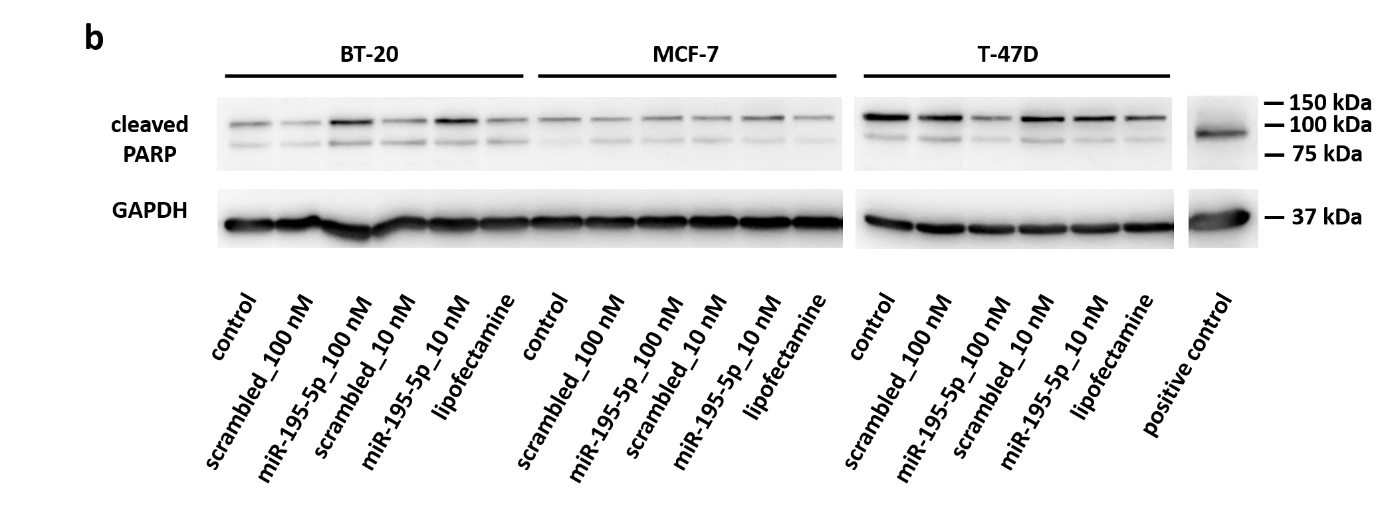
**

**Supplementary Figure S8:** Cell viability of breast cancer cell lines treated sequentially with rabusertib and doxorubicin

Data were analyzed with unpaired Student's t-test and presented as mean ± S.D. from three independent experiments; *p < 0.05, **p < 0.01, ***p < 0.001, ns = non-significant. DOX = 25 µM doxorubicin, RAB = 1 – 15 µM rabusertib.


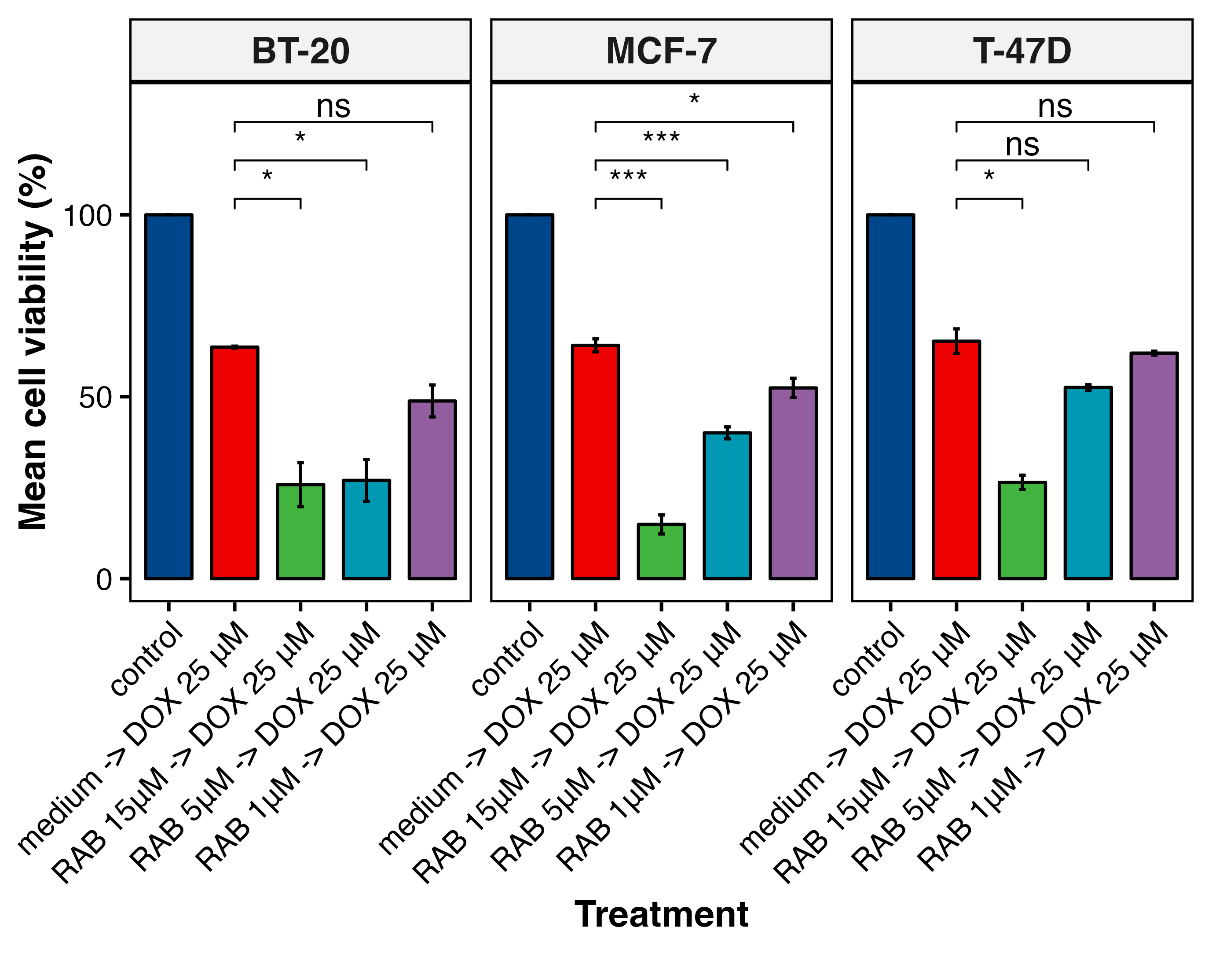

Supplement: Supplementary file 1 — Fig. S1. Clusters proposed by MCL (Markov Cluster Algorithm) cluster analysis (STRING) composed of two or more proteins, including 84 from the originally input 200 genes. Fig. S2. Correlation matrix for 19 genes selected based on the cluster analysis and literature review from the 200 potential target genes. Fig. S3. Overall survival probabilities for breast carcinoma patients stratified by subtype, therapy and expression of studied genes. Fig. S4. Relative CHEK1, CDC25A, and CCNE1 gene expression in parental breast cancer cell lines in vitro. Fig. S5. Relative changes of the CHEK1, CDC25A, and CCNE1 gene expression in breast cancer cell lines transfected with miR‐195‐5p miRNA mimics compared to control in vitro after 24 h and 48 h. Fig. S6. Protein expression of CHK1, CDC25A, and CCNE1 in breast cancer cell lines following miR‐195‐5p treatment. Fig. S7. Western blot analysis of cleaved caspase‐3 (a) and PARP (b) protein expression in breast cancer cell lines following miR‐195‐5p treatment. Fig. S8. Cell viability of breast cancer cell lines treated sequentially with rabusertib and doxorubicin. [file MOL2-19-3409-s001.docx]
